# Integrated mathematical and experimental modeling uncovers enhanced EMT plasticity upon loss of the DLC1 tumor suppressor

**DOI:** 10.1371/journal.pcbi.1013076

**Published:** 2025-05-12

**Authors:** Sebastian Höpfl, Merih Özverin, Helena Nowack, Raluca Tamas, Andrew G. Clark, Nicole Radde, Monilola A. Olayioye

**Affiliations:** 1 Institute for Stochastics and Applications, University of Stuttgart, Stuttgart, Germany; 2 Stuttgart Research Center Systems Biology, University of Stuttgart, Stuttgart, Germany; 3 Institute of Cell Biology and Immunology, University of Stuttgart, Stuttgart, Germany; 4 University of Tübingen, Center for Personalized Medicine, Tübingen, Stuttgart, Germany; Georgia Institute of Technology and Emory University, UNITED STATES OF AMERICA

## Abstract

Epithelial-mesenchymal transition (EMT) plays an essential role in embryonic development, wound healing, and tumor progression. Partial EMT states have been linked to metastatic dissemination and drug resistance. Several interconnected feedback loops at the RNA and protein levels control the transition between different cellular states. Using a combination of mathematical modeling and experimental analyses in the TGFβ-responsive breast epithelial MCF10A cell model, we identify a central role for the tumor suppressor protein Deleted in Liver Cancer 1 (DLC1) during EMT. By extending a previous model of EMT comprising key transcription factors and microRNAs, our work shows that DLC1 acts as a positive regulator of TGFβ-driven EMT, mainly by promoting SNAIL1 expression. Our model predictions indicate that DLC1 loss impairs EMT progression. Experimental analyses confirm this prediction and reveal the acquisition of a partial EMT phenotype in DLC1-depleted cells. Furthermore, our model results indicate a possible EMT reversion to partial or epithelial states upon DLC1 loss in MCF10A cells induced toward mesenchymal phenotypes. The increased EMT plasticity of cells lacking DLC1 may explain its importance as a tumor suppressor.

## Author summary

In our study, we investigate the epithelial-mesenchymal transition (EMT), a crucial process for understanding tumor progression, metastasis, and drug resistance. EMT involves complex interactions at the RNA and protein levels, leading to significant changes in cell behavior.

We focus on the role of the tumor suppressor protein Deleted in Liver Cancer 1 (DLC1) during EMT. Using breast cancer cells, we combined cellular assays with mathematical modeling to extend an existing EMT model to include DLC1. Our findings reveal that DLC1 acts as a positive regulator of EMT by promoting the expression of the transcription factor and EMT master regulator SNAIL1. When DLC1 is lost, progression of the process is impaired, and cells exhibit a partial EMT phenotype. Reversion from a completed EMT is typically impossible because cells express self-stimulatory growth factors in the mesenchymal state. Interestingly, our model also suggests that losing DLC1 in mesenchymal cells could lead to a reversion to partial or epithelial states.

Our work highlights the central role of DLC1 in cellular plasticity, underscoring its significance as a tumor suppressor. By making these findings accessible, we aim to contribute to a broader understanding of EMT and its implications for cancer research and treatment.

## Introduction

The epithelial-mesenchymal transition (EMT) is a fundamental physiological process during early development, adult tissue regeneration, and wound healing, while also playing a crucial role in pathophysiological processes such as cancer metastasis. During EMT, epithelial cells lose their characteristic features, such as apical-basal polarity and stable cell-cell junctions, while acquiring a motile phenotype and the ability to remodel the extracellular matrix, facilitating migration and invasion. In the context of cancer, these properties enable metastatic cells to leave the primary tumor site, infiltrate the circulatory system, and ultimately colonize distant organs [1–3].

The key players regulating and executing the EMT transcriptional program are well characterized. The cellular process can be triggered by various external cues, with transforming growth factor beta (TGFβ) being the most common initiator [1,4]. Downstream of TGFβ receptor signaling, the global gene expression program is regulated by the EMT Transcription Factors (EMT-TFs) snail family transcriptional repressor 1 (SNAIL1) and zinc finger E-box binding homeobox 1 (ZEB1). Their expression is regulated by two microRNA (miR) families, miR-34 and miR-200, forming two double-negative feedback loops in the network. SNAIL1 and ZEB1 repress the expression of epithelial markers, including E-cadherin (E-cad), while upregulating mesenchymal markers such as N-cadherin (N-cad). This regulation is termed “cadherin switch”, resulting in decreased cell-cell adhesion and increased cell motility [1,3,5].

Importantly, cells can simultaneously display both epithelial and mesenchymal markers, giving rise to the so-called partial EMT phenotype (P-state). The P-state comprises a broad spectrum of highly plastic intermediate cell states ranging between the fully epithelial (E-state) and mesenchymal (M-state) phenotypes [1,6]. Several studies have shown that partial EMT states increase cellular plasticity in cancer-related processes. Highly plastic partial EMT cells can possess stem-like characteristics, develop drug resistance, avoid immune surveillance, and adapt to harsher environments in circulation and distant organs. Cancer cells in the P-state exhibit higher tumorigenicity in *vivo* and show a preference towards collective migration/invasion, a strategy proposed to be necessary for the formation of metastases *in vivo.* Following metastatic dissemination, the reverse process, mesenchymal-epithelial transition (MET), promotes metastatic outgrowth [3,6].

Given the complexity of the EMT program, which involves processes that rely on interconnected feedback and feedforward loops and unfold over different time scales, mathematical modeling has proven to be a powerful tool for gaining insights into the characteristics of EMT and MET. The CBS model describes EMT by two successive transitions, from the E- to the P-state and from the P- to the M-state. These transitions are controlled by the SNAIL1/miR-34 and ZEB1/miR-200 feedback loops, which are characterized by a sigmoidal response with on-off behavior. While the first switch in the CBS model is reversible, the M-state becomes irreversible due to the activation of autocrine TGFβ expression. Therefore, investigating the circumstances that allow MET to occur is particularly interesting to understand how invasive cells revert to epithelial phenotypes when colonizing distant organs [7,8].

In addition to canonical SMAD signaling triggered by TGFβ, several non-canonical SMAD-independent pathways contribute to the EMT program. These include the Mitogen-activated Protein Kinase (MAPK), Janus Kinase, and Signal Transducer and Activator of Transcription (Jak/STAT), and Rho GTPase pathways, amongst others [9,10]. The latter orchestrate the major cytoskeletal rearrangements characteristic of EMT/MET, with RhoA, Rac, and Cdc42 being best studied. There is thus extensive crosstalk between canonical TGFβ receptor and Rho signaling at different stages of EMT, which are relevant to the metastatic properties of cancer cells [11]. The aberrant Rho activation observed in different cancers is primarily mediated by altered expression of Rho regulators, namely the Guanine nucleotide Exchange Factors (GEFs) and GTPase Activating Proteins (GAPs). While GEFs promote GTP loading of the GTPase, thereby leading to activation, GAPs negatively regulate Rho signaling. In this context, the GAP protein Deleted in Liver Cancer 1 (DLC1), with specificity mainly for RhoA and, to a lesser extent Cdc42, has been established as an important tumor suppressor [12–14]. Gene deletion and downregulation of DLC1 gene expression or protein levels frequently occur in many different types of cancers, including those of the breast, and such alterations have been shown to promote migration and invasion *in vitro*, and tumor formation and metastasis *in vivo.* Although DLC1 has been identified in a ZEB1/YAP target gene set [15], the precise role of DLC1 in EMT has not yet been investigated.

Here, we explore the role of DLC1 during EMT through a combined experimental and modeling approach. By integrating DLC1 into the CBS model in a data-driven manner, we can predict the consequences of DLC1 loss at different points of the EMT process. We show that DLC1 downregulation leads to changes in the expression levels of the master TFs SNAIL and ZEB1 and to an enrichment of subpopulations with more epithelial/partial properties during EMT and MET, suggesting a central role of DLC1 in shaping cellular plasticity.

## Results

### DLC1 functions as a positive regulator of EMT TFs in TGFβ-induced MCF10A cells

**Fig 1:**
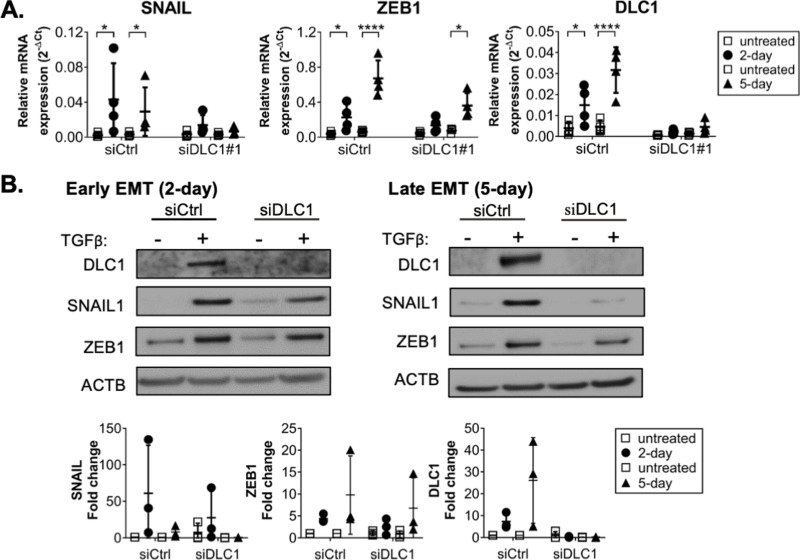
DLC1 functions as a positive regulator of EMT TFs in TGF **β-induced MCF10A cells.** **A.** mRNA expression levels of DLC1, SNAIL1, and ZEB1 at early (2-day) and late (5-day) EMT time points measured by QPCR. Data represents the mean+SD of four biological replicates. **B.** Protein expression levels measured by Western blot. Quantified fold change for three biological replicates. One-way ANOVA was performed for untreated-TGFβ comparisons with Sidak’s multiple comparison test (ns not significant, *P ≤ 0.05, ** P ≤ 0.01, *** P ≤ 0.001). The p-values, including the additional siCtrl-siDLC1 comparisons, were listed in S1 Table.

To unravel the potential role of DLC1 in the induction and progression of EMT, we utilized the human mammary MCF10A cell line as a well-established EMT model that transitions from an epithelial to a mesenchymal phenotype in response to exogenous TGFβ stimulation. We established that after 2 days of TGFβ treatment, cells exhibited early EMT properties, characterized by a substantial upregulation of *snail1* mRNA levels, in line with the known function of SNAIL1 in governing the first switch from E-state to P-state. After 5 days of TGFβ treatment, the model system displayed late-EMT characteristics, with *snail1* levels starting to decrease while *zeb1* levels progressively increased to enable the second switch towards the M-state (Fig 1A) [7,8]. The protein abundances of SNAIL1 and ZEB1 followed a similar trend with their transcript levels (Fig 1B). Notably, along with the main EMT TFs SNAIL1 and ZEB1, DLC1 was strongly upregulated in response to TGFβ on mRNA and protein levels at both time points (Fig 1A and 1B). To address the role of DLC1 in the context of EMT induction (“early EMT”, after 2 days of TGFβ treatment) and progression (“late EMT”, after 5 days of TGFβ treatment), we transfected the cells with a DLC1-specific siRNA 24 hours prior the TGFβ stimulation (siDLC1). Interestingly, compared to the control siRNA setting (siCtrl), DLC1 depletion significantly perturbed the TGFβ-induced SNAIL1 upregulation. The effect on ZEB1 was similar, though less prominent (Fig 1A and 1B), indicating that despite SNAIL1 downregulation in DLC1-depleted cells, SNAIL1 nevertheless reached the required threshold level to permit the ZEB1 upregulation associated with EMT progression. We obtained consistent results using a second independent siRNA silencing DLC1 (S1A and S1B Fig).

An extended CBS model integrating DLC1 captures EMT behavior in MCF10A cells

**Fig 2:**
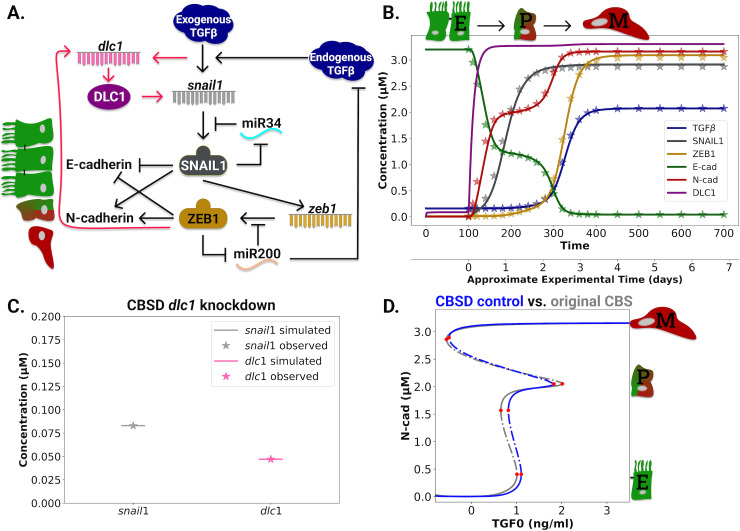
The Cascading Bistable Switch DLC1 (CBSD) model explains the EMT behavior of MCF10A cells under control and *dlc1* knockdown conditions. **A.** Interaction graph of the CBSD model. Interactions of the original CBS model are indicated with black arrows. Additional regulations through the integration of DLC1 are marked with red arrows. **B.** Fit of the CBSD model trajectories (solid lines) to the CBS model’s simulated data (stars). *dlc1* data in the siCtrl was included relative to the *zeb1* simulated data. The model was equilibrated without exogenous TGFβ stimulation for 100 timesteps and afterward stimulated with 10 ng/ml exogenous TGFβ. **C.** Fit of the CBSD model to the experimental data under *dlc1* knockdown. The stars represent the mean observed fold-changes of *dlc1 and snail1* upon *dlc1* knockdown at day 5 (S2 Fig), and the lines represent the respective model simulation values. **D.** Exogenous TGFβ (TGF0) versus N-cad bifurcation of the CBSD model under control condition (blue line) compared to the bifurcation diagram of the original CBS model (grey line).

Our experimental data focusing on perturbations of DLC1 levels during TGFβ-induced EMT in MCF10A cells suggests a positive regulation between DLC1 and the expression of *snail1* and *zeb1*, assigning a central role of DLC1 during EMT (Fig 1). As DLC1 was identified as a YAP/ZEB target gene [15], we hypothesized that DLC1, SNAIL1, and ZEB1 interact in a positive feedback loop. To explore this hypothesis, we chose a computational approach based on the qualitative Cascading Bistable Switch (CBS) model of Tian et al. [7] (Fig 2A, black arrows). Simulation of the CBS model with 10 ng/ml exogenous TGFβ stimulation shows a two-step transition from the E- to the M-state (Fig 2B, stars). The first step is activated by SNAIL1, increasing N-cad and decreasing E-cad to intermediate levels (P-state). Simultaneously, SNAIL1 activates ZEB1 via *zeb1* transcriptional activation, triggering the second switch to high N- and low E-cad levels (M-state).

We extended the CBS model with DLC1 and refer to the extension as the Cascading Bistable Switch DLC1 (CBSD) model (Fig 2A, red arrows). Compared to the original CBS model, the CBSD model contains an additional positive feedback loop comprising DLC1, SNAIL1, and ZEB1. The extension obeys that (i) EMT dynamics is preserved in the control condition where DLC1 is present (siCtrl) and (ii) the extended model captures the behavior observed under *dlc1* silencing conditions. As DLC1 was identified as a ZEB/YAP target gene [15], we included a transcriptional regulation of ZEB1 on *dlc1*. Further, our data indicates an influence of DLC1 upstream of *snail1* via transcriptional regulation. The observed increase in DLC1 in early EMT suggests that exogenous TGFβ also activates *dlc1* transcription independent of SNAIL1. To investigate TGFβ-dependent *dlc1* activation experimentally, we inhibited non-canonical TGFβ signaling, including the MAPK pathway, by MEK inhibition (S1C Fig). Since the increase in DLC1 upon TGFβ stimulation was abrogated under this condition, we concluded that the MAPK signaling pathway regulates DLC1 expression in response to TGFβ (S1C Fig). Hence, we included a direct regulation of TGFβ onto *dlc1* in the CBSD model.

The CBSD model was calibrated to the simulated time course data of the CBS model and the qPCR data after five days of TGFβ stimulation (Fig 1A). To include the *dlc1* silencing data, the observed mRNA fold changes of *snail1* and *dlc1* were converted to concentrations relative to control *snail1* concentrations (S1 Appendix). A stable *dlc1* knockdown was assumed for the investigated period. In addition, we used simulated data with 1.5 ng/ml TGFβ for model calibration (S3A Fig), which allows an accurate detection of the location of the partial state (Fig 2D). Details of the model and the fitting are described in Methods and Supplementary information (S1 Appendix) on the CBSD model. We performed a local sensitivity analysis of the CBSD model under *dlc1* knockdown for the *dlc1* and *snail1* parameters (S5 Fig). N-cadherin as model output was most sensitive to changes in the parameters kd_s_, J2_s_, kd_D_, and k_knockdown_, while changes in the parameters J2_d_, k_d2_, and k0_d_ only had a small effect.

The CBSD simulation in the control condition mimics the simulated CBS data (Fig 2B). Detailed control condition fits for all species can be found in S3B Fig. In particular, the bistable character of the transition is preserved. The DLC1 course is dominated by the direct exogenous TGFβ activation, leading to a rapid and immediate DLC1 increase after TGFβ stimulation. Transcriptional activation of DLC1 via ZEB1 is neglectable, as *dlc1* does not further increase when *zeb1* levels rise. The observed transcriptional changes in *dlc1* and *snail1* upon *dlc1* silencing were stable for the investigated time (S2 Fig) and well captured by the CBSD model (Fig 2C). A detailed fit for the CBSD model under *dlc1* silencing can be found in S4 Fig.

The bifurcation diagram of N-cad versus exogenous TGFβ (TGF0) (Fig 2D) in the CBSD control condition shows three stable fix point branches corresponding to the E, P, and M states. Since the fix point branch of the P state is bounded by two saddle node bifurcations, both of which appear at positive TGF0 values, the transition from E (low N-cad branch) to P (middle branch) is reversible upon TGF0 removal. The second transition to the M state (upper branch) is irreversible, in agreement with the CBS model of Tian et al. [7] In summary, the CBSD model can mimic EMT dynamics after TGFβ stimulation and integrates changes in mRNA amounts upon silencing of *dlc1*.

### Experiments validate a role for DLC1 in EMT progression

**Fig 3:**
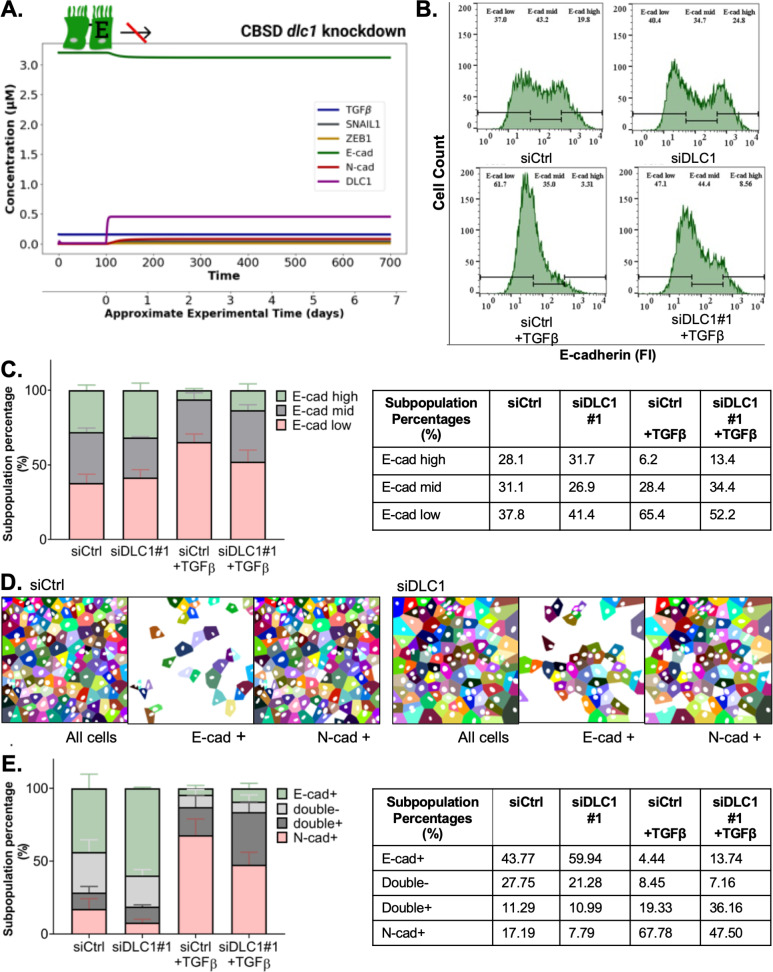
DLC1 loss impairs EMT progression of MCF10A cells. **A.** *dlc1* knockdown prevented EMT in simulations with the CBSD model. Cells with *dlc1* knockdown maintained high E-cad and low N-cad concentrations with 10 ng/ml TGFβ stimulation *in silico.* **B, C.** Loss of DLC1 impaired the TGFβ-induced decrease in E-cad expression in early EMT (3-day). The total abundance of E-cad in MCF10A cells was measured by flow cytometry, gated to divide the cell population into three subpopulations: E-cad_low_, E-cad_mid_, and E-cad_high_ **B.** The histograms are representative of three biological replicates. **C.** Average subpopulation percentages in each gate, depicted as E-cad_high_, E-cad_mid_, and E-cad_low_ subpopulation percentages in green, gray, and red, respectively (left) and table listing corresponding average values (right). P-values for siCtrl untreated vs. TGFβ and siDLC1 untreated vs TGFβ comparisons were calculated using two-way ANOVA in GraphPad Prism for all the subpopulations: E-cad low (0.0004 and 0.1556), E-cad mid (0.6299 and 0.4216) and E-cad high (0.0027, 0.0103) (n = 3). P-values for siCtrl TGFβ vs. siDLC1 TGFβ for all the subpopulations: E-cad low (0.0667), E-cad mid (0.6051) and E-cad high (0.4445) (n = 3). **D, E.** Immunofluorescence (IF) imaging to classify cells in M-, E- and P-state (N-cad + ; E-cad + ; double+ or double-, respectively) using plasma membrane staining of E-cad and N-cad markers. **D.** Representative processed IF images in late-EMT used for classification based on EMT marker expression, showing the segmentation of cell nuclei and the predicted pseudo-colored cell masks showing “positive” cells in each for individual channel (E-cad+ and N-cad+). The corresponding IF images are provided in the S8 Fig. **E.** Stacked bar graphs for the average subpopulation percentages of three biological replicates for siDLC1#1(left) and table listing corresponding average values (right). Subpopulation percentages were calculated by the total number of cells per condition. (500-1000 cells/condition, right). Two-way ANOVA (Tukey’s multiple comparisons test for siCtrl vs. siDLC1#1 in each subpopulation) were calculated for the untreated condition: E-cad+ (0.0470), N-cad+ (0.0357), double+ (0.9976) and double- (0.5277); and for +TGFβ condition: E-cad+ (0.5824), N-cad+ (0.0023), double+ (0.0455) and double- (0.9705).

To confirm and further understand the role of DLC1 in EMT, we used the CBSD model to simulate the system’s response to TGFβ treatment under *dlc1* knockdown conditions (Fig 3A). The model suggests that *dlc1* silencing prevents EMT induction, with cells trapped in the E-state (S7 Fig). While SNAIL1, DLC1, and ZEB1 protein concentrations slightly increase, and E-cad slightly decreases after TGFβ stimulation, the extent is too low for the transition to the P-state. These simulations predict that DLC1 is essential for the cells to undergo EMT.

To test these predictions experimentally, we performed flow cytometry of MCF10A cells to measure total E-cad expression as a marker of the epithelial state on a single-cell level. We identified three subpopulations in early EMT: E-cad low, E-cad mid, and E-cad high (Fig 3B). The gates were defined to correspond to the two peaks in the control cells, E-cad low and E-cad high, and the middle slope between the two peaks as E-cad mid. In contrast to our *in silico* model, which started with an entirely epithelial population, the untreated MCF10A cell population displayed heterogeneity in E-cad expression, with approximately 40% of cells in the E-cad low subpopulation. This indicates a mixed initial population of cells within a spectrum of epithelial to mesenchymal phenotypes. Upon TGFβ treatment, the E-cad high subpopulation was reduced from 28.1% to only 6.2% in the control setting and from 31.7% to 13.4% in *dlc1* knockdown MCF10A cells. At the other end of the spectrum, consistent with the model which proposed MCF10A cells to be in the transition between the P- and the M-state around this time (Fig 2B), we found the cells almost entirely in the E-cad mid and E-cad low subpopulations. In the control condition, the E-cad low subpopulation expanded by an average of 27.6%, while the E-cad mid population decreased by an average of 2.7%, showing that most cells strongly downregulated E-cad expression in response to TGFβ. By contrast, only 11% of the cells shifted to the E-cad low gate when DLC1 was lost, while the E-cad mid population increased by an average of 7.5%. Thus, by switching from the E-/P-state to the P-/M-state, the data suggests that EMT progression still occurred when DLC1 was depleted (Fig 3C). Taken together, our experimental data shows that upon *dlc1* knockdown, more cells remained in the E-/P-state when stimulated with TGFβ. In the control setting, most cells transitioned to the P-/M-state, suggesting a predominant partial EMT phenotype in cells with DLC1 loss at this time point.

To analyze the effect of DLC1 on EMT dynamics at the “late” timepoint (day 6 -/ + TGFβ), we employed immunofluorescence imaging as a method preserving cell morphology and used the cadherin switch as a parameter to classify cells. As expected for the epithelial state, untreated cells presented packed cobblestone morphology with high E-cad expression (green) on their plasma membrane (S8A Fig). TGFβ treated cells, on the other hand, were packed relatively loosely and disorganized, with E-cad being replaced by N-cad expression (red) on the plasma membrane (S8B Fig). In the case of *dlc1* knockdown, E-cad expression appeared to be retained in a higher proportion of cells. Employing an unbiased and automated immunofluorescence analysis, MCF10A nuclei were segmented based on the DAPI staining, while cell boundaries were predicted using the Voronoi tessellation based on nuclear centroids (Fig 3D). For each channel, cells displaying IF signals above the background threshold were assigned as “positive” for the respective marker, enabling a classification into four distinct categories: E-cad + , N-cad + , double positive (double+), or double negative (double-), where E-cad + , N-cad + , and double+ cells represented E-state, M-state, and P-state, respectively (Fig 3D). Double- cells could be considered a subpopulation of the P-state, possibly captured in a “naïve” transition state [16]. We observed that in late-EMT, most of the cells were sorted as N-cad + , thus in the M-state, and the rest were mainly double + , therefore in the P-state (Fig 3E), in line with the corresponding simulations (Fig 2B). When DLC1 was lost, the N-cad+ subpopulation was significantly reduced, while the number of double+ cells increased, displaying a stronger shift to the partial EMT phenotype. The results were comparable using an independent siRNA targeting DLC1 (S8C Fig). In sum, both in early-EMT and late-EMT, we demonstrated through independent experimental approaches that a clear shift towards the E-/P-states occurred for *dlc1* knockdown populations, consistent with our model predictions.

### DLC1 loss alters MET dynamics in MCF10A upon TGFβ washout

**Fig 4:**
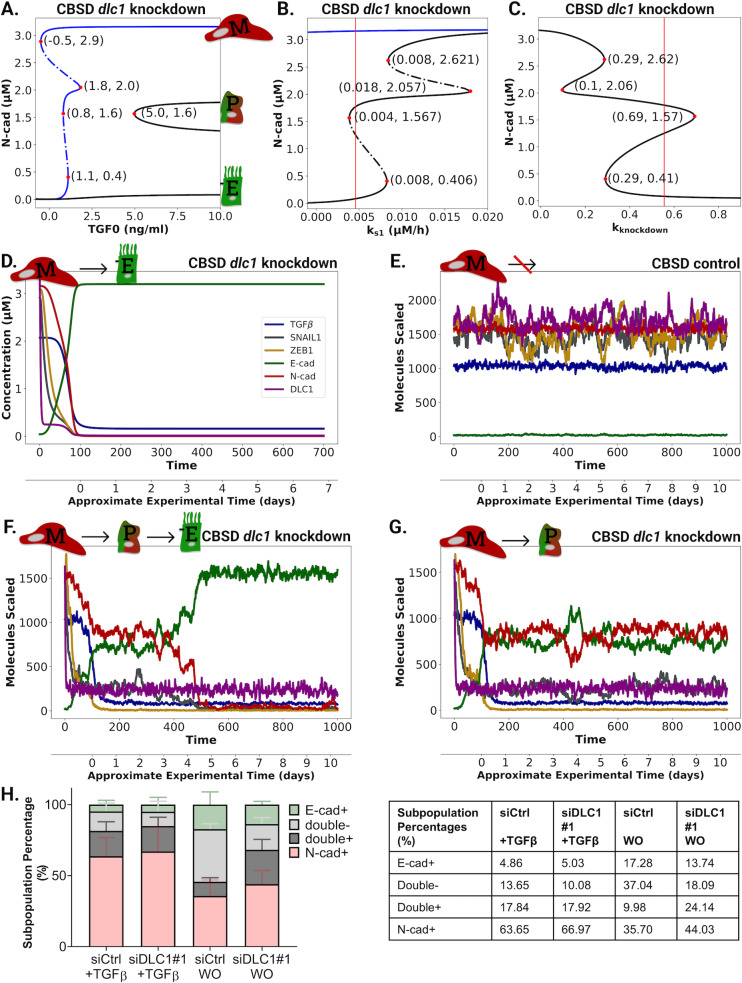
Stochasticity enables MET in *dlc1* knockdown simulations. **A., B. & C.** TGF0, k_s1_, and k_knockdown_ versus N-cad bifurcations in the CBSD model under *dlc1* knockdown. For a direct comparison, the CBSD *dlc1* knockdown bifurcation is shown in black, and the CBSD control setting is shown in blue. The bifurcations with k_s1_ and k_knockdown_ were made with 10 ng/ml exogenous TGFβ. Vertical red lines are ML estimates of parameters. **D.** Deterministic simulation of the CBSD model under *dlc1* knockdown without exogenous TGFβ. The simulation starts in the M-state. **E.** Stochastic simulations of the CBSD model in the control condition with 10 ng/ml exogenous TGFβ starting in the M-state. **F. & G.** Stochastic simulations of the *dlc1* knockdown CBSD model with 10 ng/ml exogenous TGFβ show MET to the E (left) and P (right) states. **H.** Stacked bar graphs for the average subpopulation percentages of three biological replicates for siDLC1#1 (left) and table listing corresponding average values (right). Subpopulation percentages are calculated by the total number of cells per condition. (500-1000 cells/condition, right). Two-way ANOVA (Tukey’s multiple comparisons test for siCtrl vs. siDLC1#1 in each subpopulation) were calculated for M-state (+TGFβ) condition: E-cad+ (0.9994), N-cad+ (0.9018), double+ (0.9997) and double- (0.6932); and for washout (WO) condition: E-cad+ (0.7755), N-cad+ (0.5310), double+ (0.0009) and double- (0.0006).

EMT supports the initial stages of metastatic dissemination of cancer cells, whereas its reversal is crucial for metastatic colonization [17]. In the control cells, the mesenchymal state is irreversible (siCtrl; Fig 2D). As our data suggests a central role of *dlc1* in regulating EMT, we investigated the reversibility of the M-state under *dlc1* silencing conditions. First, we analyzed the qualitative CBSD model behavior of DLC1-depleted cells using bifurcation analysis. Bifurcation parameters were (i) exogenous TGFβ (Fig 4A), (ii) the TGFβ dependent *snail1* transcription rate $k_{s1}$ (Fig 4B), and (iii) the *dlc1* knockdown strength (Fig 4C). The CBSD model has a stable fix point branch with low N-cad concentration (representing the E-state), persisting for 0–10 ng/ml exogenous TGFβ stimulation (Fig 4A). Starting at 4.97 ng/ml exogenous TGFβ, two distinct stable partial states with medium N-cad concentrations appear. This finding aligns with recent results, showing that EMT can have multiple intermediate partial states [8,16,18–21].

Using $k_{s1}$ as a bifurcation parameter, the CBSD model has five saddle-node bifurcations, indicating that an increased TGFβ dependent *snail1* transcription rate could compensate for *dlc1* downregulation (Figs 4B and S5E). Stable fix point branches for the E and P states exist at the $k_{s1}$ Maximum Likelihood (ML)-estimate (0.0047 µM/h, Fig 4B vertical red line). For lower $k_{s1}$ values, the E-state is the only stable state due to insufficient transcriptional activation of *snail1*. For $k_{s1}$ above 0.008 µM/h the E fix point branch terminates and is replaced by an M-state branch with high N-cad. Increased TGFβ-dependent *snail1* transcription leads to the coexistence of stable P- and M-state branches (0.008 < $k_{s1}$ < 0.018 µM/h), showing the importance of this rate for the overall EMT. In comparison, in the CBSD model control condition, the M branch is the only stable fix point branch for the $k_{s1}$ ML-estimate (S9A Fig). This M branch in the CBSD model control setting is unaffected by an increase in $k_{s1}$. The $k_{knockdown}$ bifurcation diagram shows two stable P-states and one E-state in a knockdown range between 29% to 69% (Fig 2C). The ML estimate of $k_{knockdown}$ with 0.56% is in the center of this region. Without exogenous TGFβ stimulation, only the E-state is stable at the $k_{s1}$ and $k_{knockdown}$ ML estimates (S9B and S9C Fig). In short, the bifurcation analysis indicates a plastic P-state and a stable E-state when DLC1 is silenced.

*In silico*, mesenchymal cells reverted to the epithelial state after *dlc1* knockdown. Simulations starting in the M-state showed an immediate and complete reversal to the E-state after *dlc1* knockdown (Fig 4D). This reversion indicates that DLC1 is crucial for reaching and maintaining the model’s M-state. In cells with limiting DLC1 levels, the extent to which SNAIL1 is activated is insufficient for maintaining the M-state, even with high exogenous TGFβ concentrations.

Single-cell experiments revealed considerable molecular and phenotypic heterogeneity in the MCF10A cell population. There are various sources causing heterogeneity in cell populations, such as stochastic effects during cell growth and partitioning of biomolecules [22], transcriptional and translational bursts [23], and extrinsic and intrinsic noise. In addition, *dlc1* and *snail1* concentrations in the cells are considerably low (< 0.1 µM), making them prone to stochastic effects. Stochastic effects might lead to different MET outcomes, even with ongoing exogenous TGFβ stimulation. To investigate the robustness of the CBSD model against fluctuations and stochastic effects, we performed stochastic model simulations using Gillespie’s algorithm. For details, see Supplement”Rate propensity conversion” (S1 Appendix) and S10 Fig. The results of this stochastic simulation were confirmed by a RACIPE analysis [24], which incorporates heterogeneity by random parameter variation (S11 Fig). For a detailed RACIPE method description and comparison of the experimental and RACIPE results, see S1 Appendix.

Even by explicitly modeling stochastic effects, the control cells did not undergo MET in the continuous presence of exogenous TGFβ (Fig 4E). In a simulation of a population of 1000 cells, all cells (100%) remained in the M-state. However, under *dlc1* knockdown, a stochastic simulation of a population of 1000 cells revealed that 46.3% of the cells reverted to the E-state, and 53.7% reverted to the P-state. Exemplary trajectories are shown in Fig 4F and 4G. After approximately 10 experimental days, the CBSD model predicts that none of the cells in the population remain in the M-state after *dlc1* knockdown.

To validate the model predictions in the context of MET, we downregulated DLC1 after 7 days of TGFβ treatment by siRNA transfection of the cells and either continued the treatment or withdrew exogenous TGFβ for another three days. We employed the same immunofluorescence analysis to classify the different cell states. Approximately 80% of the cells were categorized as N-cad + , while the remaining were either double+ or double-, indicating that the entire cell population did not reach a stable M-state experimentally (S12A Fig). We found that *dlc1* knockdown in this quasi-mesenchymal state did not influence the EMT marker dynamics in the presence of exogenous TGFβ (Fig 4H). When TGFβ was withdrawn, both control and *dlc1* knockdown cells exhibited MET, as the percentage of E-cad+ cells increased while N-cad+ decreased. The experimental endpoint of 3 days after knockdown corresponded to the P-state in the stochastic simulation timeline (Fig 4F). Under *dlc1* knockdown, we found a significantly larger double + , and reduced double- subpopulation compared to the control setting, indicating a delay in EMT dynamics. Subpopulation percentages were consistent using the second siRNA (S12B and S12C Fig).

In summary, our deterministic and stochastic model predictions showed that DLC1 loss enables MET, the extent of which depends on the presence or absence of exogenous TGFβ. Experimental results confirmed that DLC1 downregulation promotes the cadherin switch in mesenchymal cells upon TGFβ withdrawal, thus potentially providing an advantage to metastatic cancer cells during colonization of distant organs.

## Discussion

Partial EMT states in cancer cells have been recognized to play a critical role in metastatic dissemination and therapy resistance, correlating with poor patient survival. Despite their clinical importance, the factors contributing to EMT plasticity are poorly understood. Here, we explored how the loss of the tumor suppressor DLC1 modulates EMT and MET processes in the TGFβ-responsive MCF10A model system. By extending the current CBS model developed by Tian et al. [7], we revealed how a putative positive feedback loop between DLC1 and the EMT-TFs SNAIL1 and ZEB1 affects EMT dynamics. The two bistable switches of the CBS model that govern the transition of cells between the E-, P- and M-states were validated by quantitative measurements [8]. Importantly, in our CBSD model comprising DLC1, both experiments and model hypotheses agree well in showing that DLC1 downregulation induces a considerable shift toward the epithelial state. Our study establishes an essential role of DLC1 in the dynamics of TGFβ-induced EMT and implies that cancer cells with DLC1 loss may be endowed with enhanced plasticity, favoring the survival of metastatic cells.

In line with the model predictions, experimental data confirmed that upon TGFβ treatment, *dlc1* knockdown cells exhibited partial epithelial phenotypes more prevalently. Although our initial simulations predicted cells to be trapped in the epithelial state in the absence of DLC1 resulting from low *snail* concentrations, this discrepancy can be explained by the heterogeneous nature of the MCF10A cell population in terms of EMT marker expression even prior to TGFβ treatment. Cells *in vitro* respond to multiple environmental cues, which may affect the TGFβ response. MCF10A cells are highly plastic, with E-cad and vimentin expression levels correlating with the cell density [16]. Within cell clusters, E-cad is stabilized on the cell surface, whereas single MCF10A cells lacking cell-cell contacts readily express vimentin. It is important to note that also *in vivo* solid tumors are known to display intra-tumoral heterogeneity [25]. In contrast, the mathematical model assumes that the starting cell population is fully epithelial. This heterogeneity at the single-cell level was recovered at least partially by stochastic simulations using the Stochastic Simulation Algorithm. More accurately capturing cellular heterogeneity would require defining different sources of variability, ranging from differences in the exposure to TGFβ to different protein amounts in individual cells and stochastic fluctuations in chemical reaction kinetics [26]. The advantage of the CBSD model is its ability to capture the process with a minimum set of variables and explain the relationship between DLC1 and the core EMT regulators while mirroring the trends depicted by our MCF10A cell model.

Similarly, the heterogeneous nature of the MCF10A cell population can also explain the discrepancies we observed in the modeling of MET. To capture the latter process *in silico,* the *dlc1* knockdown was performed in the M-state, while in our experimental setting, the cell population had not reached a fully mesenchymal state at the time of the *dlc1* knockdown/TGFβ withdrawal. Deterministic simulations of the CBSD model starting in the mesenchymal state together with exogenous TGFβ withdrawal predict that the cells with DLC1 loss undergo MET and eventually return to the E-state, Moreover, even in the presence of exogenous TGFβ, stochastic simulations show transitions to either the partial or the epithelial states upon DLC1 loss. Also experimentally, we observed significant changes in the dynamics of cell surface marker distributions during MET. Although the loss of DLC1 in the quasi-mesenchymal MCF10A cells did not show an effect in the presence of TGFβ, upon its withdrawal we found more *dlc1* knockdown cells expressing both E- and M- state markers, indicating that they regained E-cad expression on the plasma membrane promptly upon TGFβ washout, while in the control setting, MCF10A cells tended to lose N-cad expression first, transiting into an initial double negative state. Along these lines, Hong et al. [16] observed that MCF10A cells lost their initial phenotypes (E or M) first and then gained their destination phenotypes (M or E), transitioning through a “naïve” state that might serve to erase memories of old lineages, thus promoting differentiation plasticity.

Retaining E-cad expression along with mesenchymal markers was observed in leading cells during collective invasion [27]. A similar scenario may underlie the pro-migratory behavior of DLC1-negative cancer cell lines, including breast cancer [28–30]. Interestingly, DLC1 downregulation abrogated the negative regulation of ectopic E-cad on RhoA activity, thereby allowing anchorage-independent growth and migration in lung cancer [31]. Hence, higher E-cad expression and, as a result, a partial EMT phenotype upon DLC1 loss may contribute to cellular plasticity, in normal development and metastatic diseases. This notion is supported by recent findings in neural crest cells, in which DLC1 was identified as an intermediate cell state marker playing a functional role in delamination and migration [32]. Moreover, the metastasis suppressor role of DLC1 is potentially linked to its ability to modulate cellular plasticity, has been established *in vivo* in independent studies. Overexpression of DLC1 in the metastatic M4A4 breast cancer cell line inhibited pulmonary metastasis formation in an orthotopic mouse model, while primary tumor growth remained unaffected [16]. Restoration of DLC1 expression in TP53^−/−^;RAS^V12^ hepatoma mouse models reduced not only primary tumor growth after subcutaneous injection and orthotopic transplantation, but also lung metastasis formation after tail-vein injection [33,34].

Mechanistically, TGFβ is known to activate SMAD-dependent and independent pathways during EMT progression, including the MAPK cascade. ERK activation contributes to TGFβ-induced EMT through a set of target genes involved in disassembling adherens junctions and cell motility [35]. Interestingly, MEK inhibition suppressed TGFβ-induced DLC1 expression in our *in vitro* MCF10A model. The contribution of DLC1 to EMT dynamics, particularly in the early phases of the TGFβ response, can thus be explained by the association of DLC1 with the MAPK pathway, prior to the later transcriptional activation of DLC1 by YAP/ZEB1. Beyond cooperating with MAPK, the Rho pathway plays a complex role in TGFβ signaling. While TGFβ stimulation triggers local RhoA degradation through Par6-Smurf1, contributing to the destabilization of tight junctions, RhoA activation is simultaneously required for actin reorganization during EMT [36]. Loss of DLC1 could thus affect different subcellular Rho pools in a distinct manner. Moreover, longer exposure to TGFβ alters the expression of the Rho GTPases and their regulators. Thus, the timing of the DLC1 loss would further determine the phenotypic outcome based on the balance of the Rho signaling network in different cell states.

The core EMT circuit has already been extended by various factors, and crosstalk effects with the NF-kB pathway have been studied. For example, Jolly et al. [37] propose the stabilization of partial EMT states by GRHL2 and OVOL, which both form a mutually inhibitory transcriptional feedback loop with ZEB1. From a modeling viewpoint, adding positive feedback loops onto the core network can lead to multi-stability. Multiple positive feedback can thereby reveal the existence of multiple distinct partial states and alter parameter and input ranges in which those states stably co-exist. Here, modeling of these stabilizing components revealed that the partial states could be transiently stable and are connected to aggressive tumor progression. These results indicate that the behavior, the dynamic properties of the EMT core network, and its robustness are highly context-dependent and influenced by interactions and cross-talks with various other factors and pathways. DLC1 might be an accelerator of the EMT process by promoting a quicker transition through more hybrid microstates [38] in MCF10A cells. Consequently, DLC1 loss could be equivalent to retaining GRHL2 and OVOL during EMT, in that they act as critical molecular brakes, preventing EMT completion and contributing to the stabilization of partial states. Taken together, we believe that combining experiments and modeling enables a network viewpoint on EMT dynamics and can reveal molecular mechanisms of EMT control in the cellular context.

## Limitations of the study

Considering the heterogeneity of cell populations, future studies on the single-cell level should address how the duration of TGFβ exposure and specific signaling profiles define precise cellular phenotypes. In the case of the MAPK pathway, this can be achieved by employing genetically encoded fluorescent biosensors, enabling imaging of ERK activation dynamics in individual cells and correlating these with distinct (partial) EMT states [39,40]. Moreover, the current CBSD model version only displays the activation of *snail1* and *zeb1* upon TGFβ induction. However, experimental data indicate that *snail1* activation is transient, as *snail1* levels decline in late EMT after the *zeb1* module has been activated. Transient TGFβ input courses could mimic such behavior.

## Materials and methods

### Mathematical Modeling

Details of the mathematical model can be found in the supplementary information (S1 Appendix). Further, the CBSD model in the SBML [41] format and the Jupyter notebooks to perform the deterministic and stochastic simulations can be found on DaRUS (https://doi.org/10.18419/DARUS-4240). To make also the parameter estimation reproducible and transparent, we provide a PEtab [42] file, including the CBSD model, parameters, observables, measurements, and experimental conditions. The CBSD model also uses Hill kinetics to be consistent with the structure of the CBS model. Additional interactions and equations for *dlc1* and *dlc1* have been added. The control and the *dlc1* knockdown condition were implemented via a Boolean parameter, and we assumed the fraction of *dlc1* molecules affected by the knockdown is constant, implying a constant *dlc1* knockdown efficiency in the observed concentration range of *dlc1*. We performed Markov Chain Monte Carlo sampling to constrain the posterior and performed a model reduction based on the correlations observed in the scatter plots. All parameters of the reduced model could be estimated with small uncertainties. Posterior distributions were inferred by drawing 1 million samples using the adaptive metropolis sampler of PyPESTO [43]. Burn-in samples were removed using the Gewekes test, and convergence of the chains was examined visually via the traces and the effective sample size.

Libroadrunner [44] was used to simulate the calibrated CBSD model. For the stochastic simulations, using Gillespie’s algorithm, each subreaction was explicitly included as an individual reaction. Here, a subreaction corresponds to one particular process, for example, RNA transcription, translation, and degradation are individual events. Modeling subreactions explicitly ensures they can be triggered individually and do not need to happen simultaneously.

### Model calibration

The CBSD model was calibrated to mimic the original CBS model behavior under the control condition and our qPCR data under the *dlc1* knockdown condition. Based on the qPCR data, we calculated the ratio of *dlc1* to *snail1* in MFC10A cells to be ~ 0.77 without TGFβ and ~0.99 with TGFβ. The detailed calculation can be found in the supplementary information on *dlc1* concentration calculation (S1 Appendix). This ratio was used to include *dlc1* in the CBSD control condition. The original CBS model was simulated with 10 ng/ml exogenous TGFβ to obtain sample points for the CBSD control condition (S3B Fig). The fold-changes of our qPCR data were used to calculate fitting points of *snail1* and *dlc1* under *dlc1* knockdown. Our data shows a stable *dlc1* knockdown (S2 Fig). Therefore, the fold change from control to *dlc1* knockdown of our qPCR data was applied to the steady-state concentrations of *dlc1,* i.e., the fold change was applied to the time points 180, 200, 220, 240, 260, 280, 300, 350, 400, 500, 600, 700, 800, and 1000. The same was done for the *snail1* fold change from the control to the *dlc1* knockdown condition (Fig 2C). Integrating the knockdown data over several fitting points allowed an accurate fitting of a stable knockdown to the measured fold change and improved the weighting of the experimentally observed fold change data relative to the time series data generated with the CBS model. The partial state was also included in the model calibration to reproduce the bifurcation behavior of the CBS model under control conditions. For this purpose, the original CBS model was stimulated with 1.5 ng/ml exogenous TGFβ, and the simulation points of *zeb1* and ZEB1 were included for the calibration (S3A Fig). The CBSD PEtab file on DaRUS contains a reproducible and complete documentation of the model calibration.

The CBSD model was calibrated using 15 starts of the differential evolution algorithm of SciPy [45] with a Latin hypercube-initiated population of 100 particles and subsequent polishing via the L-BFGS-B method. The model calibration was based on three of the four replicates in Fig 1. The fourth replicate’s experiment was conducted after the modeling studies were completed to provide additional data for the siDLC1#2 control and is, therefore, not part of the model calibration. The modeling data is part of the PEtab file on the DaRUS repository, which explicitly contains the first three replicates.

### Bifurcation analysis

The bifurcation analysis was performed with PyDSTool [46]. A detailed notebook, including the hyperparameters for each bifurcation, can be found on DaRUS.

### Cell culture

MCF10A cells (ATCC Cat# CRL-10317, RRID: CVCL_0598) were cultured in DMEM/F12 with GlutaMAX (Gibco) supplemented with 2% horse serum (Invitrogen), 20 ng/ml EGF (R&D), 10 µg/ml insulin (Sigma-Aldrich), 0.5 µg/ml hydrocortisone (Sigma-Aldrich) and 100 ng/ml Choleratoxin (Sigma-Aldrich). Cells were incubated in a humidified atmosphere with 5% CO_2_ at 37°C. The passage number was kept under 40. The MCF10A cells were seeded and transfected with siRNA using Lipofectamine RNAiMAX (Invitrogen) in OptiMEM (Gibco) according to manufacturer’s instructions (reverse transfection). The siRNAs used were negative control siRNA (siCtrl, ON-TARGETplus non-targeting control pool D-001810–10 (Dharmacon), siDLC1#1 (Silencer Select Custom siDLC1#97, Cat No. 4390827, Ambion by life technologies) and siDLC1#2 (Hs_DLC1 siRNA_11, Cat No. 1027417, Qiagen).

### TGFβ stimulation

For all experiments, the starting point of the experiment (t_0_) was defined as the timepoint when 10 ng/ml TGFβ (recombinant human TGFβ, PeproTech) treatment was first added to the culture. From that point onward, depending on the length of the experiment, TGFβ was replenished every second day.

For the early EMT timepoint, TGFβ treatment was started 24h after the siRNA transfection. The cells were collected for QPCR and Western blot experiments after 48 hours. Flow cytometry experiments for the early EMT time point were conducted by simultaneous knockdown and TGFβ treatment at t_0_. the cells were re-plated after 48 hours to facilitate detachment using Collagenase the following day. Just as for early EMT, in case of the late-EMT timepoint, the siRNA transfection was performed 24 hours prior to the start of the TGFβ treatment (t_0_). To maintain the efficient DLC1 knockdown throughout the experiment, the cells were collected, re-transfected and re-seeded on day 3. For QPCR and WB experiments, the cells were collected after 5 days. For the IF experiments, the cells were collected and seeded overnight on coverslips on day 5 and fixed on day 6 (approximately 16-18h after the seeding). For the M-state/washout experiments, the siRNA transfection was performed after cells were treated with TGFβ for 7 days. For the following 2 days, the cells were either cultured in the presence of TGFβ, or in its absence (washout). The cells were collected for QPCR and WB 2 days after the knockdown transfection. An additional re-seeding overnight on coverslips was included for IF experiments.

### Quantitative real-time PCR

Total RNA isolation was performed using NucleoSpin RNA Kit (Macherey-Nagel), following the manufacturer’s protocol. qRT-PCR with 100 ng template total RNA per reaction was performed using the Cfx96 device (Bio-Rad), and the Power SYBR Green 1-Step kit (Thermo Fisher Scientific) according to manufacturer’s protocol. The following primers were used: 5’-TTCCCCACAGCGCTTCCG-3’ and 5’-TCGATGGGGAACAGGAAATCTTCA-3’ for DLC1, 5’- CGAGTGGTTCTTCTGCGCTA-3’ and 5’-CTGCTGGAAGGTAAACTCTGGA-3’ for SNAI1, 5’-AAGAATTCACAGTGGAGAGAAGCCA-3’ and 5’-CGTTTCTTGCAGTTTGGGCATT-3’for ZEB1 and 5’-CTCTGCATTCTCGCTTCCTGGAG-3’ and 5’-CAGATGGATCAGCCAAGAAGG-3’ for RPLP0. The RPLP0 housekeeping gene was used for normalization and the relative gene expression levels were calculated using the 2^–ΔΔCt^ method.

### Western blot

Cell lysates were prepared using RIPA buffer (50 mMTris-HCl pH7.5, 150 mMNaCl, 1% Triton-X-100, 0.5% sodium deoxycholate, 1 mM EDTA, 0.5 mM PMSF, 0.1% SDS, 1 mM sodium orthovanadate, 10 mM sodium fluoride, and 20 mM β-glycerophosphate plus Complete protease inhibitors without EDTA (Roche)). To determine protein concentrations, Bio-Rad DC protein assay was employed. SDS-PAGE (NuPage Novex 4–12% Bis-Tris gels, Invitrogen) was performed and transferred to iBlot nitrocellulose membranes (iBlot Transfer Stack, nitrocellulose) using an iBlot device (Invitrogen). The membranes were blocked with 0.5% blocking reagent (Roche) in PBS containing 0.1%Tween-20 for 1h. The membranes incubated with primary antibodies (anti-DLC1 mouse mAb (1:500, BD Biosciences Cat# 612021, RRID:AB_399416), anti-SNAIL1 rabbit mAb (1:1000, Cell Signaling Technology Cat# C15D3), anti-ZEB1 rabbit mAb (1:1000, Cell Signaling Technology Cat# D80D3), anti-ACTB mouse mAb (1:2000, Sigma-Aldrich Cat# A1978, RRID:AB_476692), anti-pERK-p44/42 rabbit pAb (1:1000, Cell Signaling Technology Cat# 9101, RRID:AB_331646) in blocking solution supplied with 0.05% sodium azide overnight and was followed by HRP-labeled secondary antibody incubation for 1h. The protein bands were detected using ECL Western blotting substrate (Pierce) and visualized on an Amersham600 device (GE Healthcare). The quantification was conducted using ImageJ, ACTB protein levels were used for normalization.

### Flow cytometry

Cells were replated a day before flow cytometry and collected with Collagenase Type IV (Merck). The cells in suspension were fixed and permeabilized with 1:1 4% PFA + 0.1% Triton-X (5 min incubation), then washed two times and resuspended in PBA buffer (5% Horse serum+0.05% Sodium Azide in 1X PBS). Resuspended single cells were incubated with anti-E-cad primary antibody (mouse mAb (SHE78–8), Invitrogen) for 2h on ice, followed by 1h Alexa Fluor 488-coupled secondary antibody (Invitrogen) incubation. Flow cytometry was performed with the MACSQuant Analyzer (Miltenyi Biotec), and analysis was conducted using FlowJo v10 software.

### Immunofluorescence microscopy

Cells were seeded on collagen R (2 mg/ml (0.2%) in 0.1% acetic acid diluted 1:200 in 1X PBS, Serva)-coated glass coverslips a day before fixing. Cells were fixed and permeabilized in 4% PFA + 0.2% Triton-X for 15 min, followed by 15 min 150 mM glycine incubation. Coverslips were blocked with 5% goat serum (Gibco) for 30 min at room temperature. Fixed cells were stained overnight using E-cad mAb (13–5700, Cell Signaling) and N-cad mAb (sc-53488, Santa Cruz Biotechnology). Cells were incubated with Alexa Fluor 488 and 555-labeled secondary antibodies (Invitrogen) for 1 h at room temperature. Nuclei were counterstained with DAPI. Fluoromount-G (Southern Biotech) was used to mount the coverslips. Imaging was performed with Zeiss LSM980 using 20X objective, each image was a z-stack of 9 slices (total of 4 µM depth). The images were processed and analyzed using ZEN 3.6 Blue software (Zeiss) and the FIJI [47] distribution of ImageJ2. Segmentation of cell nuclear signaling and inferred cell boundaries was performed using a custom script written in the ImageJ Macro Language using the StarDist [48] and MorphoLibJ [49] plugins. 3D Z-stacks were initially compressed using a brightest point projection. Cell nuclei were then segmented using StarDist 2D, and centroid positions were extracted using MorphoLibJ. Cell boundaries were then inferred using a 2D Voronoi tessellation in FIJI. For each cell, pixels corresponding to the cell cytoplasm were defined as pixels within the cell boundary but not within the cell nucleus area. Next, the E-Cad and N-Cad images were thresholded using the FIJI implementation of the Triangle [50] method, and the total number of positive thresholded pixels for E-cad and N-cad were determined for each cell. Cells were scored as positive or negative for E-cad or N-cad using an empirical threshold of the number of positive E-Cad or N-Cad pixels kept consistent across all conditions. 500–1000 cells per condition were analyzed per experiment. The percentages of E-cad + , N-cad + , double+ and double- cells were calculated for each experiment for 3 biological replicates.

### Statistical analysis

Data were presented as mean ± standard deviation. Statistical significance was calculated using one-way ANOVA followed by Sidak’s multiple comparisons test or two-way ANOVA followed by Tukey’s multiple comparisons test GraphPad Prism 7.

## Supporting information

S1 Appendix Supplementary information on the CBSD model, rate propensity conversion, *dlc1* concentration calculation, and model order reduction. (DOCX)

S1 Table Results of the ordinary one-way ANOVA multiple comparisons of QPCR and Western Blot data. The p-values were listed for the comparisons siCtrl-siCtrl+TGFβ, siDLC1-siDLC1 + TGFβ, siCtrl-siDLC1, and siCtrl+TGFβ-siDLC1 + TGFβ. Due to the additional siCtrl-siDLC1 comparisons, the adjusted p-values may differ.
(XLSX)

S1 Fig Validation of the QPCR and Western blot with a second siRNA. **A.** mRNA expression levels of DLC1, SNAIL1, and ZEB1 at early (2-day) and late (5-day) EMT time points for siDLC1#2 measured by QPCR. Data represents mean+SD of three biological replicates. One-way ANOVA was performed for untreated-TGFβ and siCtrl-siDLC1 comparisons with Sidak’s multiple comparison test. **B.** Protein expression levels of DLC1, SNAIL1, and ZEB1 at early (2-day) and late (5-day) EMT timepoints for siDLC1#2 measured by Western blot. The blots were quantified using FIJI and normalized to ACTB levels for three biological replicates. One-way ANOVA was performed for untreated-TGFβ and siCtrl-siDLC1 comparisons with Sidak’s multiple comparison test. **C.** DLC1 protein expression upon 24h MEK inhibition using 1 µM AZD6244 measured by Western blot (n = 2 for siDLC1#1).
(TIF)

S2 Fig Validation of a stable *dlc1* knockdown throughout the experiment. **A.** mRNA expression levels of *dlc1*, at 12-hour, 1-day, 2-day, 4-day, and 5-day time points for siCtrl and siDLC1#1 measured by QPCR. Data represents one biological replicate. **B.** mRNA expression levels of *snail1*, at 12-hour, 1-day, 2-day, 4-day, and 5-day time points for siCtrl and siDLC1#1 measured by QPCR. Data represents one biological replicate.
(TIF)

S3 Fig Detailed fits of the CBSD model under the DLC1 siCtrl condition show good agreement with the sampled data. **A.** Fits of the CBSD model to ZEB1 with 1.5 ng/ml TGFβ. Fitting to a medium exogenous TGFβ concentration ensured an accurate allocation of the partial state. **B.** Fits of the CBSD model with 10 ng/ml exogenous TGFβ to sampled data points of the CBS model. *dlc1* was fitted to a stable concentration calculated relative to the stable *snail1* concentration, according to our qPCR data.
(TIF)

S4 Fig Fit of the CBSD model under *dlc1* knockdown. The stars represent expected stable fold-changes of *dlc1 and snail1* upon *dlc1* knockdown between approximate days 2 and 7.
(TIF)

S5 FigLocal sensitivity analysis of the CBSD model under *dlc1* knockdown for the *dlc1* and *snail1* parameters.Each parameter was varied in 10000 equally spaced steps ±30% around its maximum likelihood value. The CBSD model under *dlc1* knockdown was simulated for each sample, and the sensitivity is shown in the variation of the N-Cad concentration (red). The N-Cad trajectory of the maximum likelihood parameter sample is shown in black as a reference.(TIF)

S6 FigUncertainty of the CBSD model parameters.**A.** Scatterplots reveal correlations in the newly introduced parameters. There for *snail1*, $k_{s1}:J1_{s}$ and $k_{s2}:J2_{s}$ are correlated. For *dlc1*, $k_{d2}:J2_{d}$ are correlated, while $k_{d1}$ and $J1_{d}$ are well constrained. **B.** Marginal distributions of the reduced model (without parameter correlation) from MCMC sampling. All parameters have low and normal shaped marginals with narrow credibility intervals (see S1 Appendix, S1A Tab). **C.** MCMC traces of the (reduced) CBSD model. The traces show converged chains for all parameters with 50,000 burn-in samples and an Effective Sample Size (ESS) of 32,432. All parameter values are shown on the log scale.(TIF)

S7 Fig EMT-state correlation matrix between k_knockdown_ and exogenous TGFβ (TGF0). This matrix shows the EMT state after approximately 5 experimental days using the indicated parameters to simulate the CBSD model. Green, grey, and red colors denote the E-, P-, and M-states, respectively.
(TIF)

S8 Fig Immunofluorescence images for late-EMT timepoint and validation with siDLC1#2. **A.** Immunofluorescence (IF) images using plasma membrane staining of E-cad (green) and N-cad (red) markers for untreated siCtrl (top) and its representative classification images (bottom). **B.** IF images using plasma membrane staining of E-cad (green) and N-cad (red) markers for TGFβ treated siCtrl, siDLC1#1 and siDLC1#2 (top) and their representative classification images (bottom) in late-EMT using a FIJI script (N-cad + ; E-cad + ; double+ or double-). **C.** Stacked bar graphs for the average subpopulation percentages of three biological replicates for siDLC1#2 (left). Subpopulation percentages are calculated by the total number of cells per condition. (500–1000 cells/condition, right). Two-way ANOVA (Tukey’s multiple comparisons test for siCtrl vs. siDLC1#2 in each subpopulation) were calculated for untreated condition: E-cad+ (0.8718), N-cad+ (0.2207), double+ (0.7395) and double- (0.9832); and for +TGFβ condition: E-cad+ (0.5340), N-cad+ (0.0016), double+ (0.0201) and double- (0.7251).
(TIF)

S9 FigAdditional Bifurcations of the CBSD model under *dlc1* siCtrl.**Vertical red lines are Maximum Likelihood (ML) estimates of estimated parameters. A.** The *dlc1* siCtrl bifurcations of exogenous TGFβ (TGF0) versus N-Cadherin of the CBS (grey) and CBSD (blue) model are identical. All three states and the bifurcation points are met. **B.** $k_{s1}$ versus N-Cadherin bifurcation of the CBSD *dlc1* siCtrl model with 10 ng/ml exogenous TGFβ. All fold-bifurcation points are below zero $k_{s1}$. **C.** $k_{s1}$ versus N-Cadherin bifurcation of the CBSD *dlc1* knockdown model without exogenous TGFβ. All bifurcation points are above the ML estimate. **D.** $k_{knockdown}$ versus N-Cadherin bifurcation of the CBSD *dlc1* knockdown model without exogenous TGFβ. **E.** EMT-state correlation matrix between $k_{knockdown}$ and $k_{s1}$. EMT state after approximately 5 experimental days using the indicated parameters for simulation of the CBSD model. Green, grey, and red colors denote the E-, P-, and M-state, respectively.(TIF)

S10 Fig Stochastic simulations of the CBSD model. **A.** The stochastic simulation of the CBSD model in the control condition shows the same qualitative 3-state EMT behavior as the deterministic simulation. The cells go from the E state via the P to the M state and are induced by 10 ng/ml exogenous TGFβ. **B.** Stochastic simulations of E-state cells with *dlc1* knockdown maintained in the E-state. No transition to the P or M state could be observed.
(TIF)

S11 Fig Subpopulation percentages of 100 000 simulated cells with 10 ng/ml TGFβ under *dlc1* knockdown and variation of all parameters. Subpopulation simulation and calculation was based on the RACIPE method and classified to E (green), P (grey), and M (red) state cells (Huang et al. 2018) [24]. For this approach, parameters were drawn randomly from a normal distribution centered around the maximum likelihood parameter and a variance of 20% of the maximum likelihood parameter value to simulate heterogeneity. The RACIPE analysis conditions mimic the experimental control and *dlc1* knockdown (kd) conditions of the flow cytometry experiments. A detailed description of the analysis and comparison to the experimental results can be found in the S1 Appendix. **A.** Subpopulation percentages for cells simulated starting in the E state. **B.** Subpopulation percentages for cells simulated starting in the M state, with ongoing exogenous TGFβ stimulation or under TGFβ washout (WO).
(TIF)

S12 Fig Immunofluorescence images for the DLC1 KD from an M-state and validation with siDLC1#2. **A.** IF images using plasma membrane staining of E-cad (green) and N-cad (red) markers for the M-state siCtrl (top) and its representative classification images (bottom). **B.** IF images using plasma membrane staining of E-cad (green) and N-cad (red) markers for the washout siCtrl, siDLC1#1 and siDLC1#2 (top) and their representative classification images (bottom) in late-EMT using a FIJI script (N-cad + ; E-cad + ; double+ or double-). **C.** Stacked bar graphs for the average subpopulation percentages of three biological replicates for siDLC1#2 (left). Subpopulation percentages were calculated by the total number of cells per condition (500–1000 cells/condition, right). Two-way ANOVA (Tukey’s multiple comparisons test for siCtrl vs. siDLC1#2 in each subpopulation) was calculated for M-state (+TGFβ) condition: E-cad+ (0.8714), N-cad+ (0.4337), double+ (0.9102) and double- (0.4130); and for washout (WO) condition: E-cad+ (0.8566), N-cad+ (0.2476), double+ (0.8653) and double- (0.0353).
(TIF)
