# Supplementary material for: Integrated mathematical and experimental modeling uncovers enhanced EMT plasticity upon loss of the DLC1 tumor suppressor
Source: PLoS Comput Biol. 2025 May 12;21(5):e1013076. doi: 10.1371/journal.pcbi.1013076 (PMC12121911; doi:10.1371/journal.pcbi.1013076)
Supplement: S1 Appendix — (DOCX) [file pcbi.1013076.s001.docx]

## S1 Appendix - Supplementary information on the CBSD model, rate propensity conversion, *dlc1* concentration calculation, and model order reduction.

### Supplementary information on the CBSD model

The following ordinary differential equation system describes the CBSD model. Changes to the CBS model of Tian et al. (1) are marked in red:

| $\frac{d\left[ T \right]}{dt}\text{=}k0_{T}\text{+}\frac{k_{T}}{\left( \frac{\left[ R200 \right]}{J_{T}} \right)^{n}\text{+}1}\text{-}kd_{T}[T]$ | Eq. 1 |
| --- | --- |
| $\frac{d\left[ s \right]}{dt}\text{=}k0_{s}\text{+}k_{s1}\frac{1}{\left( \frac{J1_{s}}{\left[ T \right]\text{+}TGF0} \right)^{n}\text{+}1}\text{+}k_{s2}\frac{1}{\left( \frac{J2_{s}}{\left[ D \right]} \right)^{n}\text{+}1}\text{-}kd_{s}[s]$ | Eq. 2 |
| $\frac{d\left[ S \right]}{dt}\text{=}k_{S}\left[ s \right]\frac{1}{\left( \frac{\left[ R34 \right]}{J_{S}} \right)^{n}\text{+}1}\text{-}kd_{S}[S]$ | Eq. 3 |
| $\frac{d\left[ R34 \right]}{dt}\text{=}k0_{34}\text{+}k_{34}\frac{1}{\left( \frac{\left[ S \right]}{J1_{34}} \right)^{n}\text{+}\left( \frac{\left[ Z \right]}{J2_{34}} \right)^{n}\text{+}1}\text{-}kd_{34}\left[ R34 \right]$ | Eq. 4 |
| $\frac{d\left[ z \right]}{dt}\text{=}k0_{z}\text{+}k_{z}\frac{1}{\left( \frac{J_{z}}{\left[ S \right]} \right)^{n}\text{+}1}\text{-}kd_{z}[z]$ | Eq. 5 |
| $\frac{d\left[ Z \right]}{dt}\text{=}k_{Z}\left[ z \right]\frac{1}{\left( \frac{\left[ R200 \right]}{J_{Z}} \right)^{n}\text{+}1}\text{-}kd_{Z}\left[ Z \right]$ | Eq. 6 |
| $\frac{d\left[ R200 \right]}{dt}\text{=}k0_{200}\text{+}k_{200}\frac{1}{1\text{+}\left( \frac{\left[ S \right]}{J1_{200}} \right)^{n}\text{+}\left( \frac{\left[ Z \right]}{J2_{200}} \right)^{n}}\text{-}kd_{200}[R200]$ | Eq. 7 |
| $\frac{d\left[ E \right]}{dt}\text{=}k_{e1}\frac{1}{\left( \frac{\left[ S \right]}{J1_{e}} \right)^{n}\text{+}1}\text{+}k_{e2}\frac{1}{\left( \frac{\left[ Z \right]}{J2_{e}} \right)^{n}\text{+}1}\text{-}kd_{e}\left[ E \right]$ | Eq. 8 |
| $\frac{d\left[ N \right]}{dt}\text{=}k_{n1}\frac{1}{\left( \frac{J1_{n}}{\left[ S \right]} \right)^{n}\text{+}1}\text{+}k_{n2}\frac{1}{\left( \frac{J2_{n}}{\left[ Z \right]} \right)^{n}\text{+}1}\text{-}kd_{n}[N]$ | Eq. 9 |
| $\frac{d\left[ d \right]}{dt}\text{=}k0_{d}\text{+}k_{d1}\frac{1}{\left( \frac{J1_{d}}{\left[ T \right]\text{+}TGF0} \right)^{n}\text{+}1}\text{+}k_{d2}\frac{1}{\left( \frac{J2_{d}}{\left[ Z \right]} \right)^{n}\text{+}1}\text{-}kd_{d}\left[ d \right]\text{-}S_{on}\cdot k_{knockdown}[d]$ | Eq. 10 |
| $\frac{d\left[ D \right]}{dt}\text{=}k_{D}\left[ d \right]\text{-}kd_{D}[D]$ | Eq. 11 |

A term for DLC1 transcriptional promotion was added to the *snail1* ODE (Eq. 2). In addition, ODEs for the *dlc1* mRNA (Eq. 10) and DLC1 protein were added (Eq. 11). The *dlc1* ODE consists of a basal, TGFβ- and ZEB1-mediated transcription, and a degradation term (Eq. 10). The knockdown of *dlc1* was implemented via the Boolean parameter S_on_, which is 0 for the wild-type and 1 for the knockdown condition. The parameter k_knockdown_ represents the knockdown strength.

To test whether all additional parameters of the CBSD model are practically identifiable, we performed Markov Chain Monte Carlo (MCMC) sampling of the posterior distribution. The sampling scatter of the model with all parameters showed a correlation between the parameter pairs k_s1_:J1_s_, k_s2_:J2_s_ and k_d2_:J2_d_ (S6A, S6B Fig). Therefore, a model reduction was performed to obtain practically identifiable parameters and to reduce the uncertainty. Here, J1_s_, J2_s_, and J2_d_ parameters were replaced with literature values, and k_d2_ was replaced with the maximum likelihood estimate of the non-reduced model.

The fits and all results were generated using the parameters of the reduced model version. All parameters of the CBSD model are well identifiable with narrow marginals (S6C Fig). The MCMC chain is converged for all parameters (S6D Fig). Maximum Likelihood (ML), Maximum A Posteriori (MAP) estimates, and 95% credibility intervals for these parameters are listed in S1A Tab.

**S1A Tab: Maximum Likelihood (ML) and 95% Credibility Intervals (CI) of the estimated CBSD parameters.** Values are obtained from MCMC sampling. A complete list of the CBSD model's parameter values can be found in the PEtab file on the DaRUS repository.

| Parameter | ML | 95% CI |
| --- | --- | --- |
| k_s1_ | 0.0045 | [0.0022, 0.0061] |
| k_s2_ | 0.0312 | [0.0281, 0.0357] |
| J1_d_ | 2.0822 | [1.6459, 2.4648] |
| k_D_ | 16.5682 | [13.4380, 21.1112] |
| k_d1_ | 0.0307 | [0.0248, 0.0345] |
| k_knockdown_ | 0.5496 | [0.4126, 0.6989] |

### Supplementary information on the rate propensity conversion

To make the Gillespie algorithm applicable, the kinetic terms of the reaction rate equations must be translated to stochastic reaction propensities. This translation includes the conversion of concentrations in mol/l to the number of molecules per cell. The reaction propensity $a_{j}$ is defined as

$$a_{j}\text{=}\kappa_{j}\cdot\prod_{i\text{=}1}^{M} \left( \frac{n_{i}!}{\beta_{ij}!\left( n_{i}\text{-}\beta_{ij} \right)!} \right)$$

with the stochastic rate constant $\kappa_{j}$ and the product of the number of molecules $n_{i}$ with their educts stoichiometric coefficients $\beta_{ij}$. The relation between the stochastic and the deterministic reaction constant is

$$\kappa_{j}\text{=}k_{j}\cdot V\cdot\prod_{i\text{=}1}^{M} \frac{\beta_{ij}!}{V^{\beta_{ij}}}\text{ }.$$

The insertion of $\kappa_{j}$ to $a_{j}$ leads to a general expression of the stochastic reaction propensity $a_{j}$ in dependence on the deterministic rate constant $k_{j}$ (2):

$$a_{j}\text{=}\frac{k_{j}}{V^{\sum_{i}^{M} {(\beta}_{ij})\text{-}1}}\cdot\prod_{i\text{=}1}^{M} \left( \frac{n_{i}!}{\left( n_{i}\text{-}\beta_{ij} \right)!} \right)$$

For zero-order reactions, the reaction propensity reads $a_{j}\text{=}k_{j}\cdot V$, for first-order reactions $a_{j}\text{=}k_{j}\cdot n_{A}$ and for second-order reactions $a_{j}\text{=}\frac{k_{j}}{V}\cdot n_{A}\cdot n_{B}$. All equations of the CBSD model are linear combinations of zero and first-order reactions. A pseudo-steady-state assumption is used for hill kinetics terms, as suggested by Hahl and Kremling (2). Therefore, the multiple fast subreactions in a hill reaction are considered as one reaction. The first-order terms were adjusted by scaling the initial concentrations of the species to get several molecules. As the probability of zero-order reactions increases with the size of a system, they are scaled with a volume factor sigma. Scaling of the initial concentrations and the volume was made with the same factor as conducted by Tian et al. (1).

Supplementary information on *dlc1* concentration calculation

As described in the methods section, we integrated *dlc1* into the CBS model relative to *snail1.* This is done by using the $2^{\text{-}\Delta C_{T}}$ method (3) and our quantitative qPCR data with which the ratio of *dlc1* to *snail1* can be calculated with and without TGFβ stimulation.

$$R\text{=}2^{\text{-}\left[ C_{q;GOI_{A}}\text{-}C_{q;GOI_{B}} \right]}$$

$$R\text{=}2^{\text{-}\left[ C_{q;dlc1}\text{-}C_{q;snail1} \right]}$$

This results in a *dlc1:snail1* ratio of 1.304 without exogenous TGFβ and 1.009 with 10 ng/ml exogenous TGFβ from the mean value of the tree measured replicates at day 5.

### Supplementary information on model order reduction

To extend the CBS model, Eq. 2 of the CBS model was extended, and two additional equations (Eq. 10 and 11) were introduced. The parameter vector to be estimated $\theta_{est}$ therefore contains the parameters $\theta_{est}\text{=}k_{s1},\text{ }k_{s2},\text{ }J1_{s},\text{ }J2_{s},\text{ }k_{d1},\text{ }k_{d2},\text{ }J1_{d},\text{ }J2_{d},\text{ }k_{D},\text{ }k_{knockdown}$. The degradation parameters are not expected to change with the model extension and are kept at their literature values.

Bayesian Inference was used to analyze if the available data contains enough information to constrain the posterior distribution. I.e. we inferred the joint posterior distribution of $\theta_{est}$ and checked if the resulting parameters are restricted to narrow and meaningful intervals. $J1_{s}$ and $J2_{s}$ were not well restricted in their marginal posteriors. This can be explained by looking into the pairwise scatter plots of the parameters. Here, $J1_{s}$ and $J2_{s}$ were correlated with their according transcription rates $k_{s1}$ and $k_{s2}$ (S6A Fig). To reduce the overall parameter uncertainty, the model was reduced by fixing $J1_{s}$ and $J2_{s}$ to the Michaelis constant of TGFβ-dependent *snail1* translation of the CBS model (1). Further, $J2_{d}$ was set equal to $J2_{s}$ and $k_{d2}$ was set to the MAP value from the Bayesian inference with all parameters.

For the CBSD model, only the reduced parameter set $\tilde{\theta}_{est}\text{=}k_{s1},\text{ }k_{s2},\text{ }k_{d1},\text{ }J1_{d},\text{ }k_{D},\text{ }k_{knockdown}$ was estimated. The Bayesian Information criterion was used to compare the reduced model against the not reduced model. The Bayesian information criterion is calculated based on the number of estimated parameters $k$, observed data points $n$ and the maximum likelihood estimate value ${L(\theta)}_{max}:$

$$BIC\text{=}k\cdot ln\left( n \right)\text{-}2ln({L(\theta)}_{max})$$

The maximum likelihood estimate was calculated by 15 runs of the scipy differential evolution algorithm with a population of 100, Latin hypercube initiation and polishing with the *L-BFGS-B* method. The estimated maximum likelihood values were almost identical, with the maximum likelihood value of the reduced model being >99,99% of the value of the not-reduced model. This shows a strong favoring of the reduced model ($k\text{=}6$) over the non-reduced model ($k\text{=}10$), as lower BIC are favorable and all other parameters except $k$ are almost identical.

**Supplementary information on the RACIPE analysis**

A RACIPE analysis (4) was performed to model the cell population heterogeneity. Computationally this was achieved by simulating 100 000 individual cells for which each parameter of the CBSD model was drawn randomly from a normal distribution centered around the maximum likelihood parameter with a variance of 20% of the maximum likelihood value. For the analysis starting in the E state, cells were simulated for 100 time steps without exogenous TGFβ to obtain the steady-state population. Afterward, cells were simulated for approximately three experimental days with 10 ng/ml TGFβ under *dlc1* knockdown and control conditions. For the analysis starting in the M state, cells were simulated for approximately 7 days with exogenous TGFβ to obtain the mesenchymal steady-state population. Afterward, cells were simulated for approximately five experimental days with and without exogenous TGFβ.

The RACIPE approach complements the stochastic simulations with a qualitative comparison of the modeling results with experimental flow cytometry data. The comparison of the RACIPE early EMT population (S11A Fig) after about three days TGFβ stimulation shows overall a good qualitative agreement with the experimental data (Fig 3C) but quantitative differences. First, the RACIPE control and *dlc1* knockdown populations show almost no heterogeneity, whereas the experimental population does. Here, the origin of our CBSD model in the CBS model can explain the missing heterogeneity, as this model forces qualitatively strong switches. In other words, the distinct EMT states are very separated, and the 20% variation is insufficient to obtain heterogeneity. With exogenous TGFβ, the agreement to the experimental data is qualitatively good even though the shift to the E state under *dlc1* knockdown is stronger in the RACIPE population.

The RACIPE population (S11B Fig) and the experimental flow cytometry population starting in the mesenchymal state (Fig 4H) show a comparable heterogeneity under control conditions with TGFβ stimulation. Under *dlc1* knockdown, the RACIPE population shows already a shift to the E and P states, while a shift in the experimental population is only observed in the washout experiments. In the washout population, the RACIPE epithelial population was larger than in the experiments. Here, the model has no double negative state and shifts this population probably to the epithelial population. The P state is the population where both E- and N-cad are present in the model with medium concentrations. Therefore, the P state in the model corresponds best to the double positive population in the experiments and the predictions agree qualitatively with an increase in this population under *dlc1* knockdown.

Overall, the RACIPE analysis could give rise to the experimentally observed heterogeneity, even if the quantitative proportions differed, which was expected due to the CBSD model’s qualitative nature.

Literature Cited

1. Tian X-J, Zhang H, Xing J. Coupled reversible and irreversible bistable switches underlying TGFβ-induced epithelial to mesenchymal transition. Biophys J 2013; 105(4):1079–89.

2. Hahl SK, Kremling A. A Comparison of Deterministic and Stochastic Modeling Approaches for Biochemical Reaction Systems: On Fixed Points, Means, and Modes. Front Genet 2016; 7:157.

3. Livak KJ, Schmittgen TD. Analysis of relative gene expression data using real-time quantitative PCR and the 2(-Delta Delta C(T)) Method. Methods 2001; 25(4):402–8.

4. Huang B, Jia D, Feng J, Levine H, Onuchic JN, Lu M. RACIPE: a computational tool for modeling gene regulatory circuits using randomization. BMC Syst Biol 2018; 12(1):74.
