# Supplementary figures and images for: Integrated mathematical and experimental modeling uncovers enhanced EMT plasticity upon loss of the DLC1 tumor suppressor
Source: PLoS Comput Biol. 2025 May 12;21(5):e1013076. doi: 10.1371/journal.pcbi.1013076 (PMC12121911; doi:10.1371/journal.pcbi.1013076)

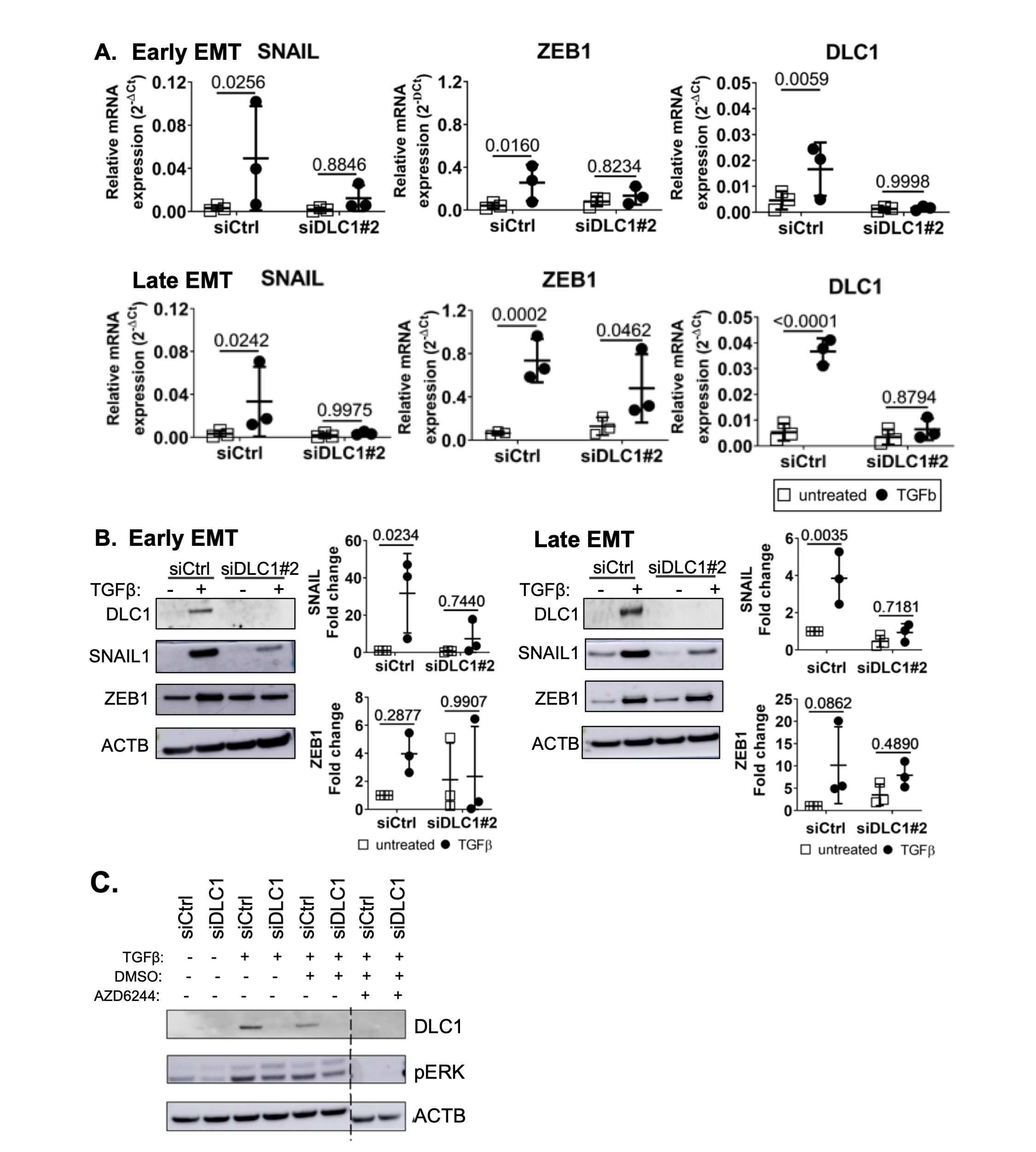

Supplement: S1 Fig — A. mRNA expression levels of DLC1, SNAIL1, and ZEB1 at early (2-day) and late (5-day) EMT time points for siDLC1#2 measured by QPCR. Data represents mean+SD of three biological replicates. One-way ANOVA was performed for untreated-TGFβ and siCtrl-siDLC1 comparisons with Sidak’s multiple comparison test. B. Protein expression levels of DLC1, SNAIL1, and ZEB1 at early (2-day) and late (5-day) EMT timepoints for siDLC1#2 measured by Western blot. The blots were quantified using FIJI and normalized to ACTB levels for three biological replicates. One-way ANOVA was performed for untreated-TGFβ and siCtrl-siDLC1 comparisons with Sidak’s multiple comparison test. C. DLC1 protein expression upon 24h MEK inhibition using 1 µM AZD6244 measured by Western blot (n = 2 for siDLC1#1). (TIF) [file pcbi.1013076.s003.tif]

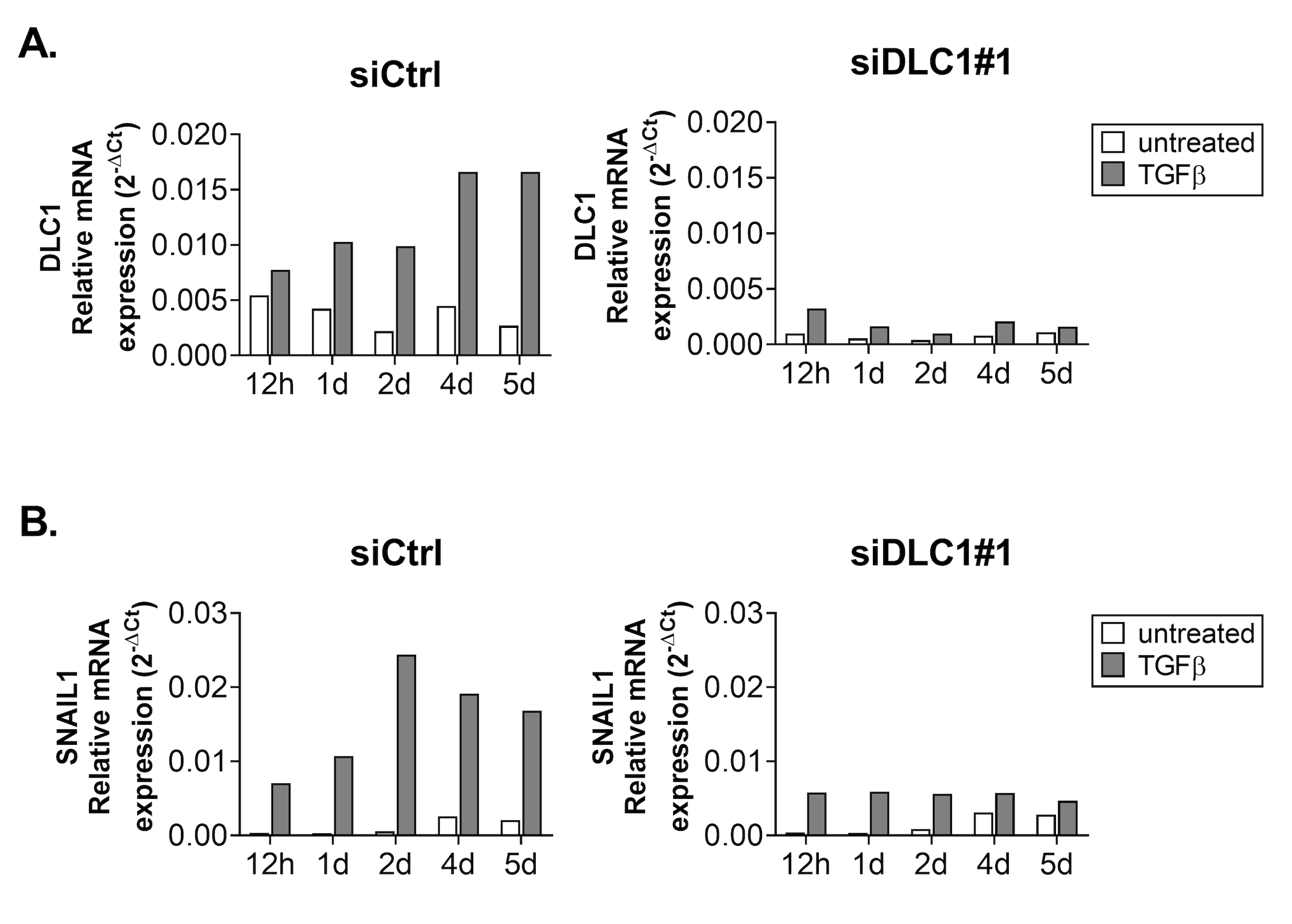

Supplement: S2 Fig — A. mRNA expression levels of dlc1, at 12-hour, 1-day, 2-day, 4-day, and 5-day time points for siCtrl and siDLC1#1 measured by QPCR. Data represents one biological replicate. B. mRNA expression levels of snail1, at 12-hour, 1-day, 2-day, 4-day, and 5-day time points for siCtrl and siDLC1#1 measured by QPCR. Data represents one biological replicate. (TIF) [file pcbi.1013076.s004.tif]

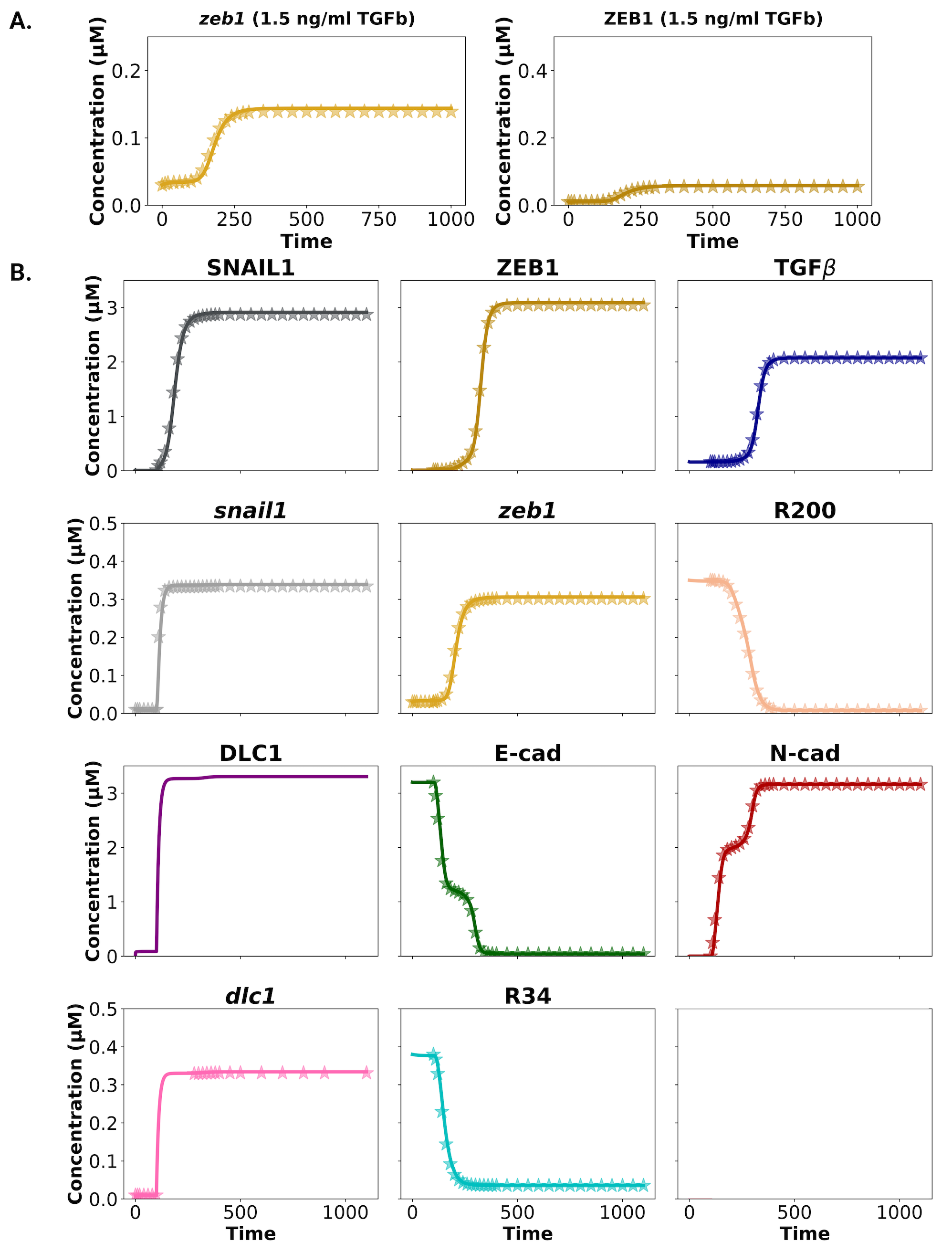

Supplement: S3 Fig — A. Fits of the CBSD model to ZEB1 with 1.5 ng/ml TGFβ. Fitting to a medium exogenous TGFβ concentration ensured an accurate allocation of the partial state. B. Fits of the CBSD model with 10 ng/ml exogenous TGFβ to sampled data points of the CBS model. dlc1 was fitted to a stable concentration calculated relative to the stable snail1 concentration, according to our qPCR data. (TIF) [file pcbi.1013076.s005.tif]

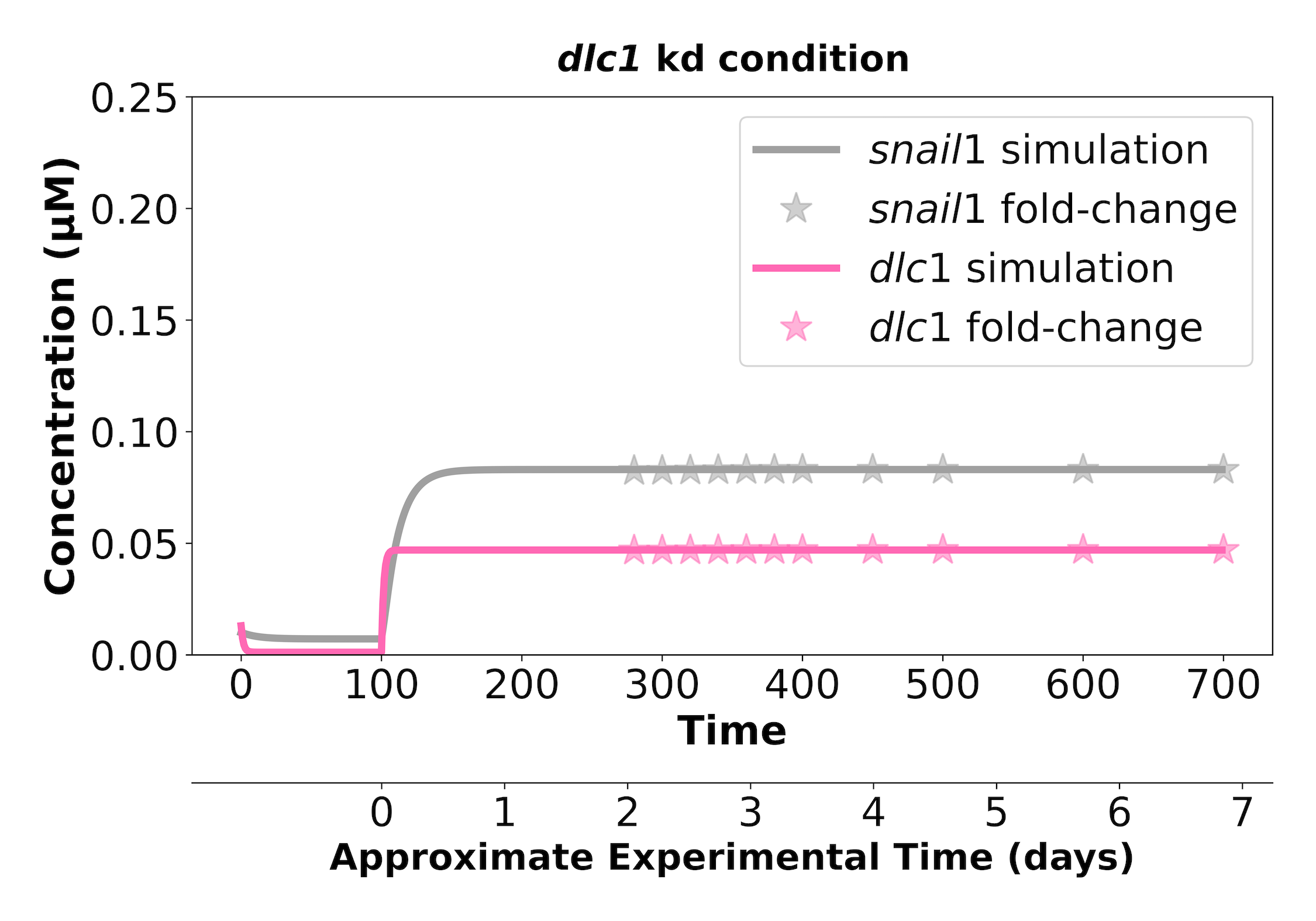

Supplement: S4 Fig — The stars represent expected stable fold-changes of dlc1 and snail1 upon dlc1 knockdown between approximate days 2 and 7. (TIF) [file pcbi.1013076.s006.tif]

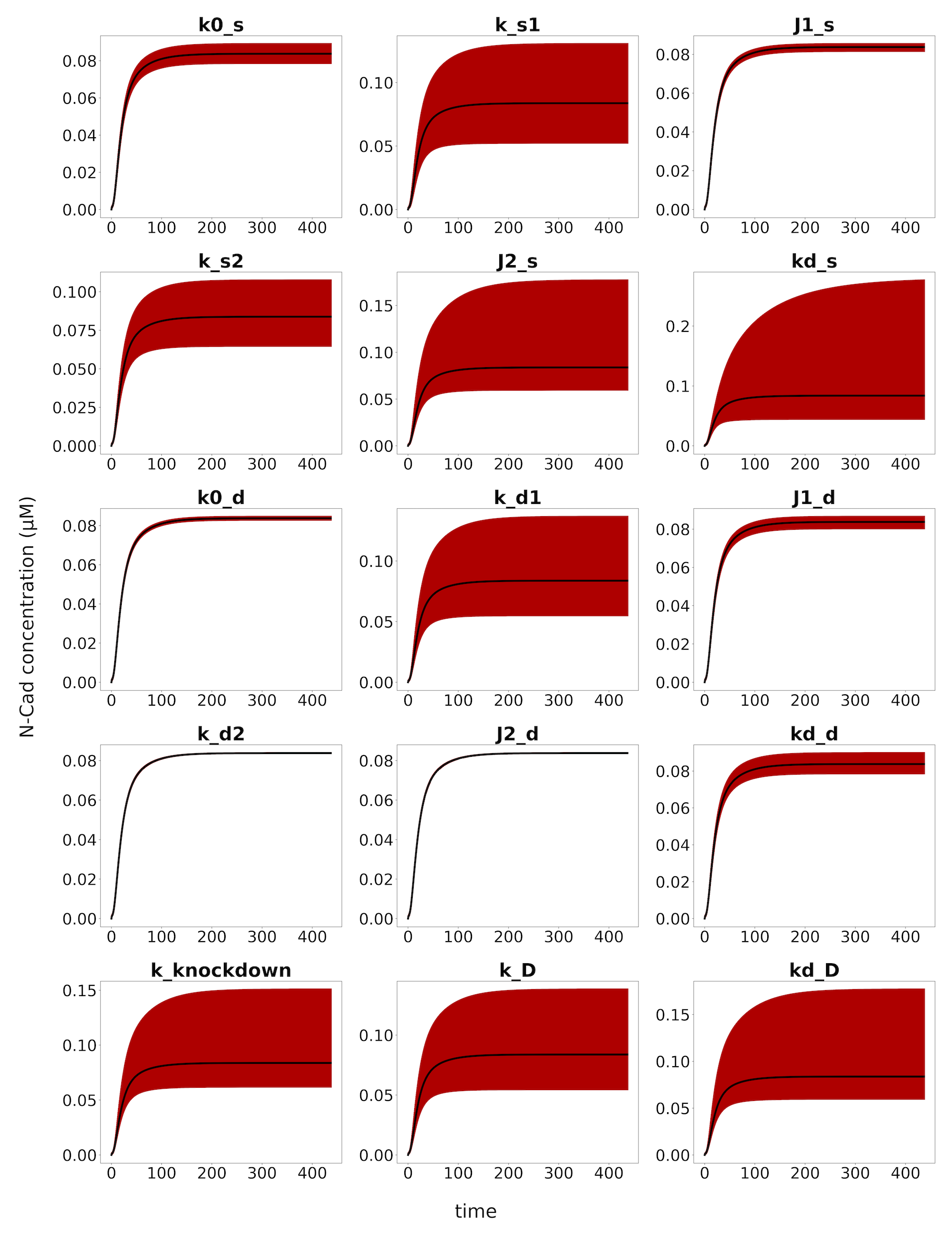

Supplement: S5 Fig — Each parameter was varied in 10000 equally spaced steps ±30% around its maximum likelihood value. The CBSD model under dlc1 knockdown was simulated for each sample, and the sensitivity is shown in the variation of the N-Cad concentration (red). The N-Cad trajectory of the maximum likelihood parameter sample is shown in black as a reference. (TIF) [file pcbi.1013076.s007.tif]

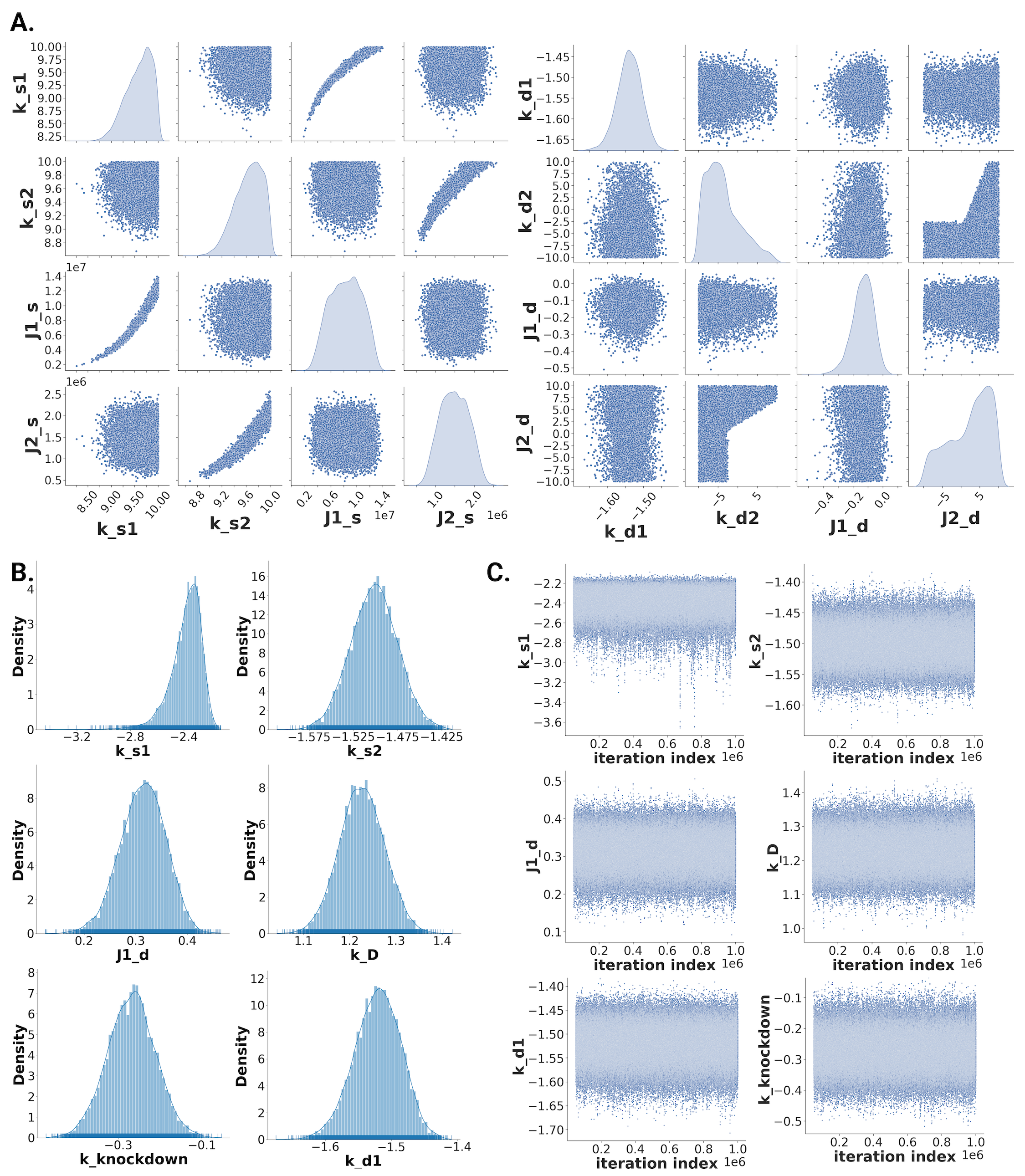

Supplement: S6 Fig — A. Scatterplots reveal correlations in the newly introduced parameters. There for snail1, ks1:J1s and ks2:J2s are correlated. For dlc1, kd2:J2d are correlated, while kd1 and J1d are well constrained. B. Marginal distributions of the reduced model (without parameter correlation) from MCMC sampling. All parameters have low and normal shaped marginals with narrow credibility intervals (see S1 Appendix, S1A Tab). C. MCMC traces of the (reduced) CBSD model. The traces show converged chains for all parameters with 50,000 burn-in samples and an Effective Sample Size (ESS) of 32,432. All parameter values are shown on the log scale. (TIF) [file pcbi.1013076.s008.tif]

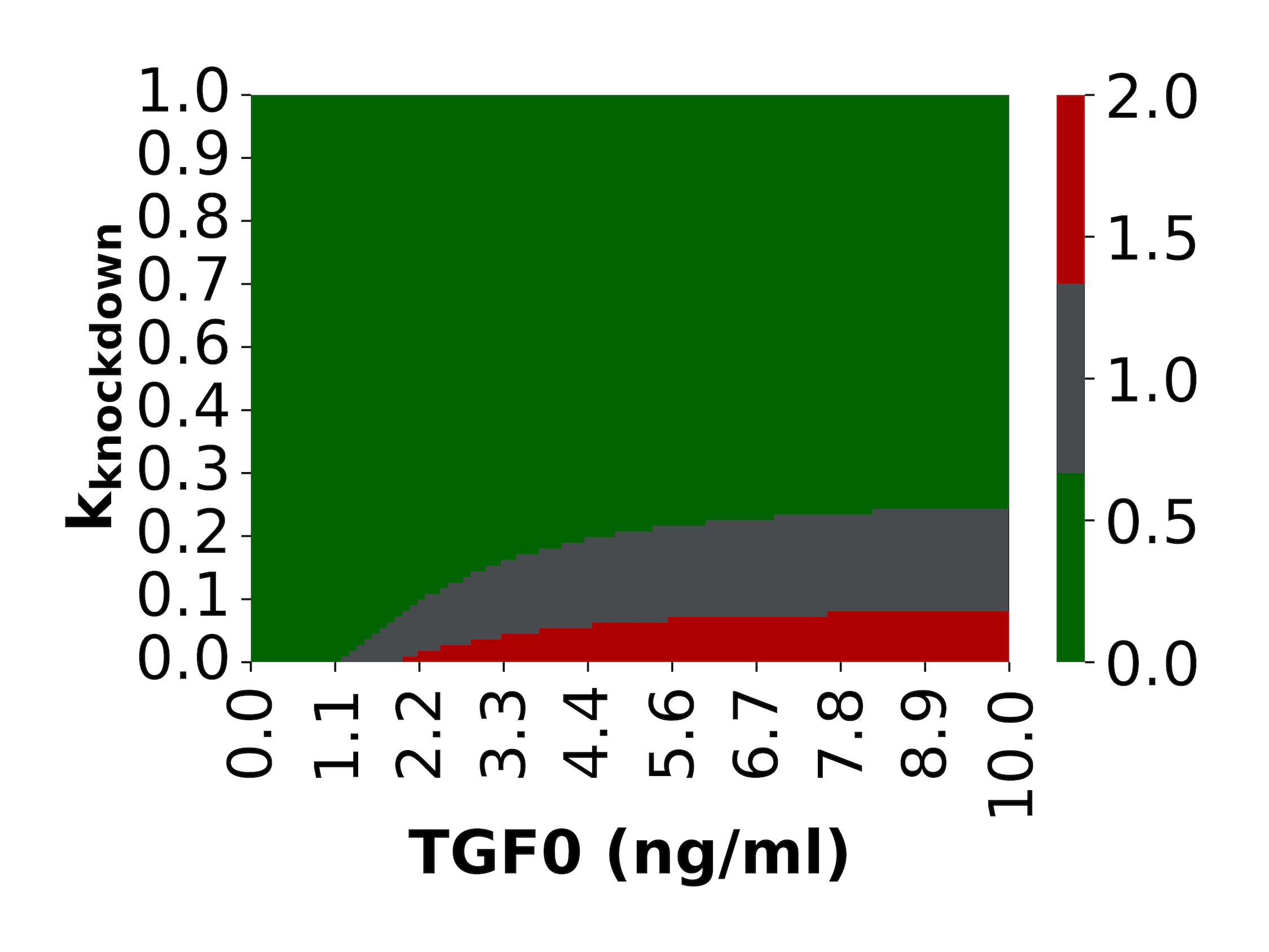

Supplement: S7 Fig — This matrix shows the EMT state after approximately 5 experimental days using the indicated parameters to simulate the CBSD model. Green, grey, and red colors denote the E-, P-, and M-states, respectively. (TIF) [file pcbi.1013076.s009.tif]

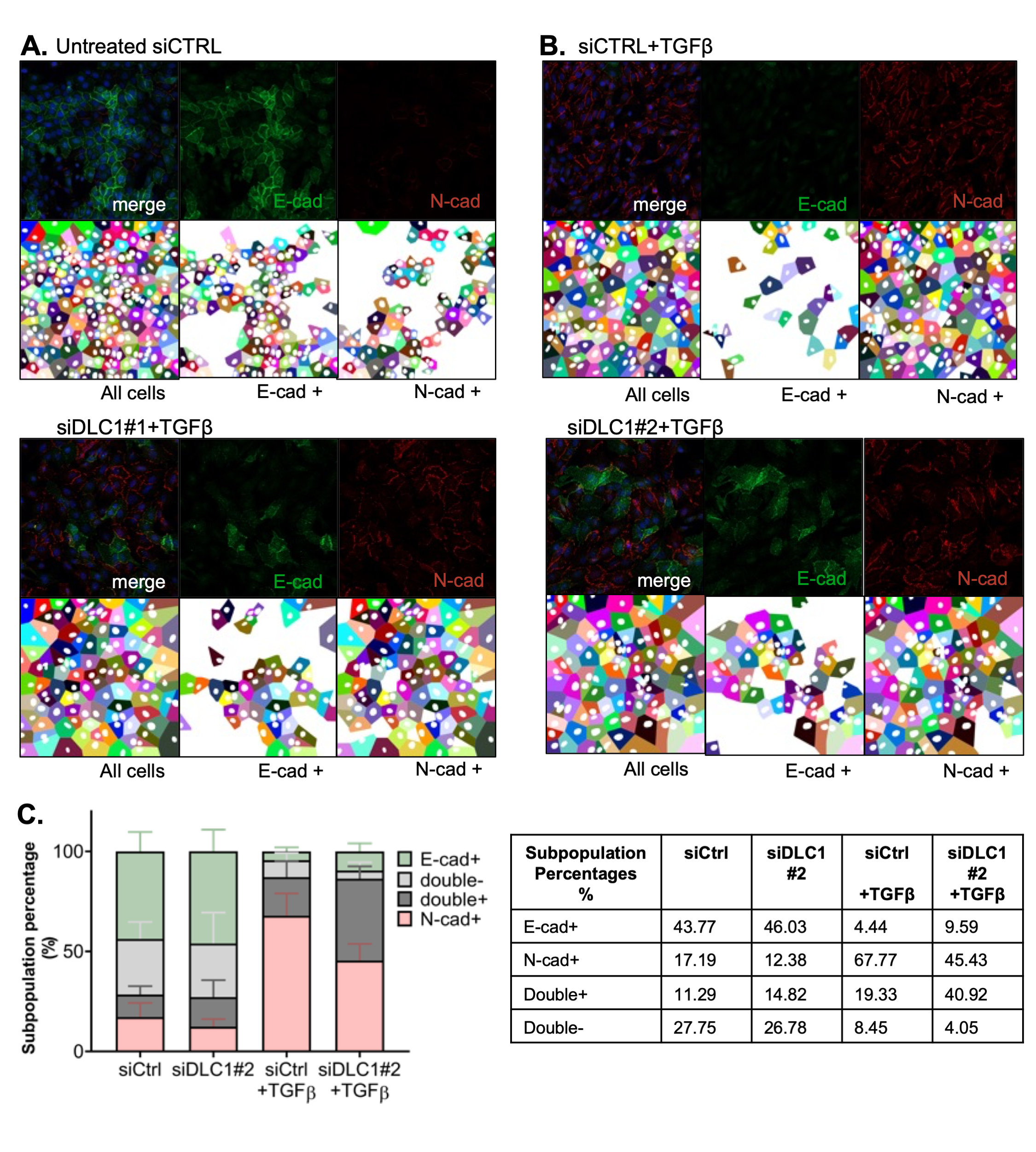

Supplement: S8 Fig — A. Immunofluorescence (IF) images using plasma membrane staining of E-cad (green) and N-cad (red) markers for untreated siCtrl (top) and its representative classification images (bottom). B. IF images using plasma membrane staining of E-cad (green) and N-cad (red) markers for TGFβ treated siCtrl, siDLC1#1 and siDLC1#2 (top) and their representative classification images (bottom) in late-EMT using a FIJI script (N-cad + ; E-cad + ; double+ or double-). C. Stacked bar graphs for the average subpopulation percentages of three biological replicates for siDLC1#2 (left). Subpopulation percentages are calculated by the total number of cells per condition. (500–1000 cells/condition, right). Two-way ANOVA (Tukey’s multiple comparisons test for siCtrl vs. siDLC1#2 in each subpopulation) were calculated for untreated condition: E-cad+ (0.8718), N-cad+ (0.2207), double+ (0.7395) and double- (0.9832); and for +TGFβ condition: E-cad+ (0.5340), N-cad+ (0.0016), double+ (0.0201) and double- (0.7251). (TIF) [file pcbi.1013076.s010.tif]

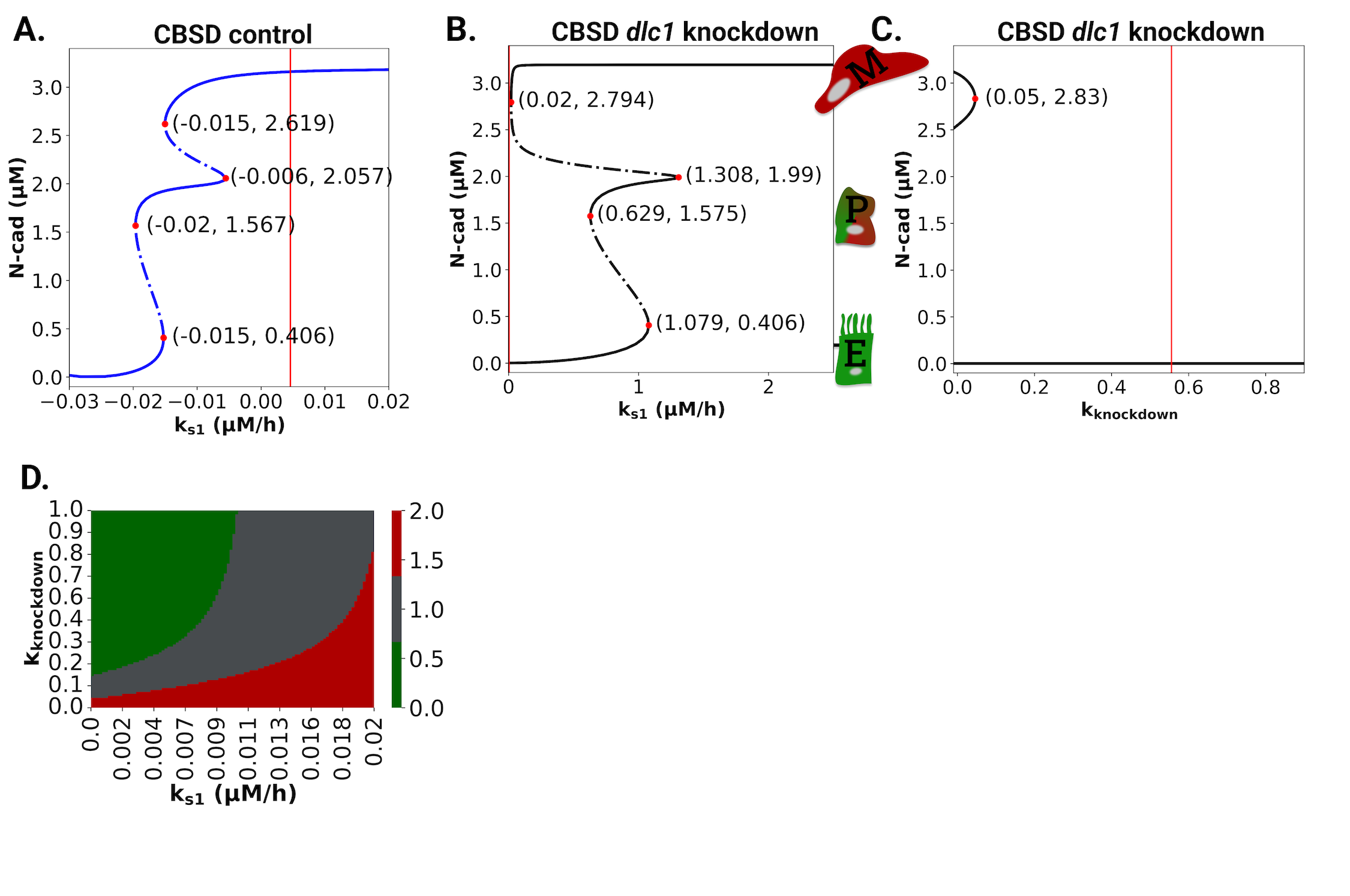

Supplement: S9 Fig — Vertical red lines are Maximum Likelihood (ML) estimates of estimated parameters. A. The dlc1 siCtrl bifurcations of exogenous TGFβ (TGF0) versus N-Cadherin of the CBS (grey) and CBSD (blue) model are identical. All three states and the bifurcation points are met. B. ks1 versus N-Cadherin bifurcation of the CBSD dlc1 siCtrl model with 10 ng/ml exogenous TGFβ. All fold-bifurcation points are below zero ks1. C. ks1 versus N-Cadherin bifurcation of the CBSD dlc1 knockdown model without exogenous TGFβ. All bifurcation points are above the ML estimate. D. kknockdown versus N-Cadherin bifurcation of the CBSD dlc1 knockdown model without exogenous TGFβ. E. EMT-state correlation matrix between kknockdown and ks1. EMT state after approximately 5 experimental days using the indicated parameters for simulation of the CBSD model. Green, grey, and red colors denote the E-, P-, and M-state, respectively. (TIF) [file pcbi.1013076.s011.tif]

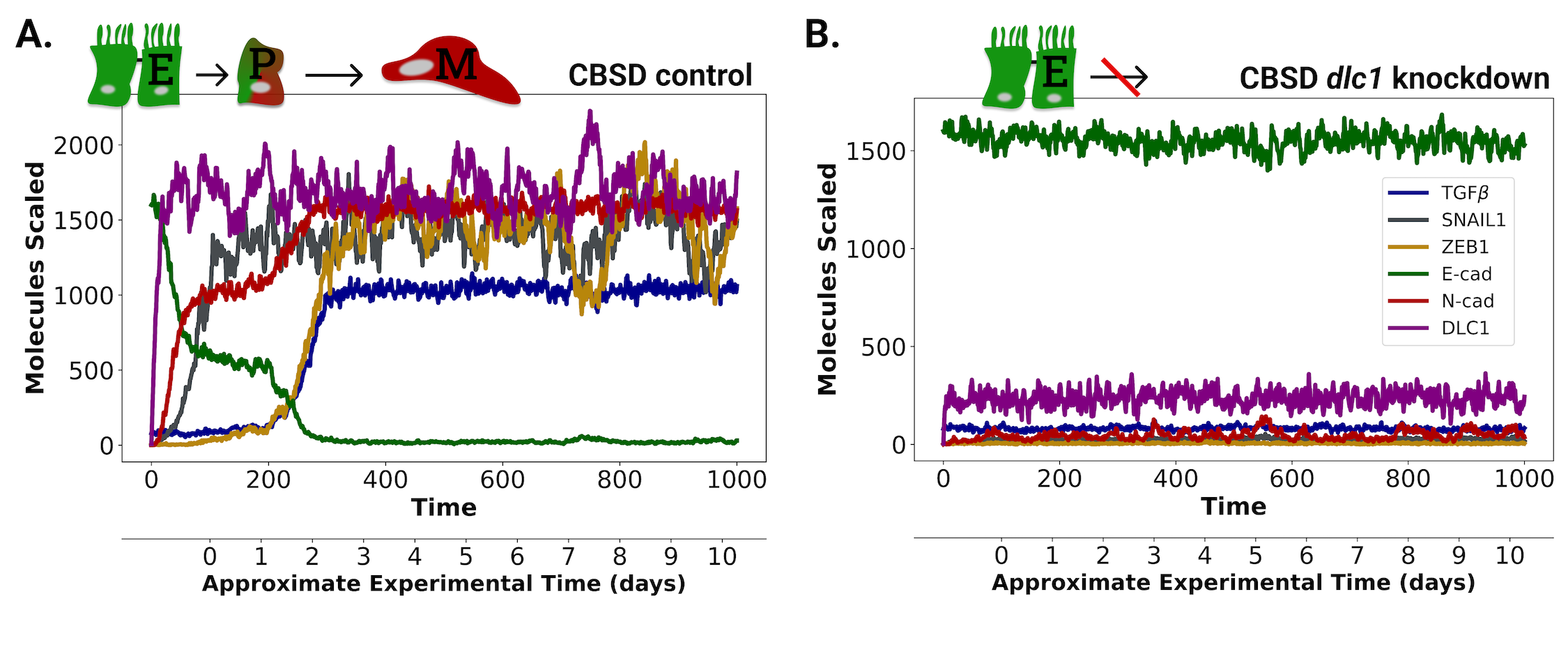

Supplement: S10 Fig — A. The stochastic simulation of the CBSD model in the control condition shows the same qualitative 3-state EMT behavior as the deterministic simulation. The cells go from the E state via the P to the M state and are induced by 10 ng/ml exogenous TGFβ. B. Stochastic simulations of E-state cells with dlc1 knockdown maintained in the E-state. No transition to the P or M state could be observed. (TIF) [file pcbi.1013076.s012.tif]

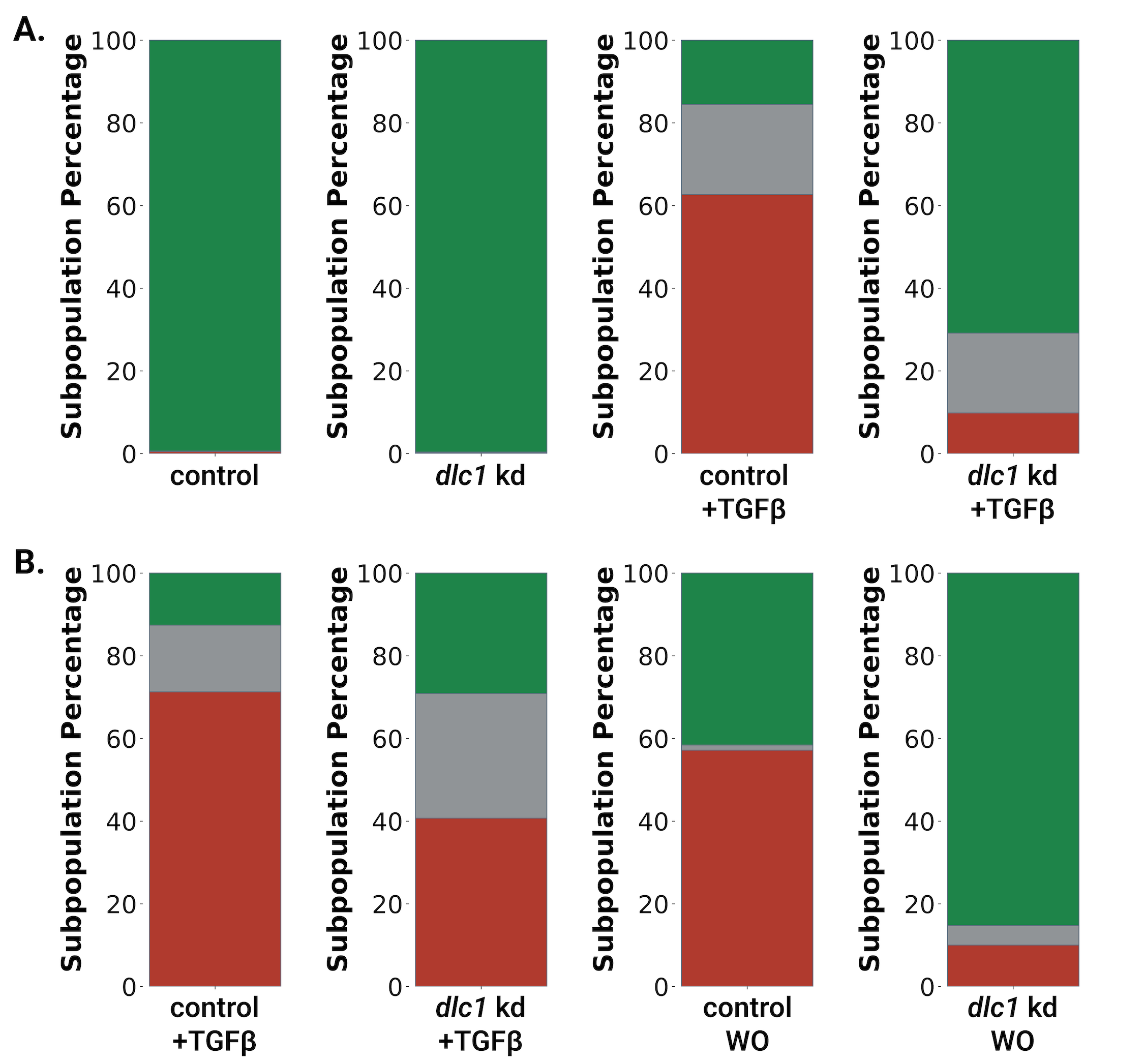

Supplement: S11 Fig — Subpopulation simulation and calculation was based on the RACIPE method and classified to E (green), P (grey), and M (red) state cells (Huang et al. 2018) [24]. For this approach, parameters were drawn randomly from a normal distribution centered around the maximum likelihood parameter and a variance of 20% of the maximum likelihood parameter value to simulate heterogeneity. The RACIPE analysis conditions mimic the experimental control and dlc1 knockdown (kd) conditions of the flow cytometry experiments. A detailed description of the analysis and comparison to the experimental results can be found in the S1 Appendix. A. Subpopulation percentages for cells simulated starting in the E state. B. Subpopulation percentages for cells simulated starting in the M state, with ongoing exogenous TGFβ stimulation or under TGFβ washout (WO). (TIF) [file pcbi.1013076.s013.tif]

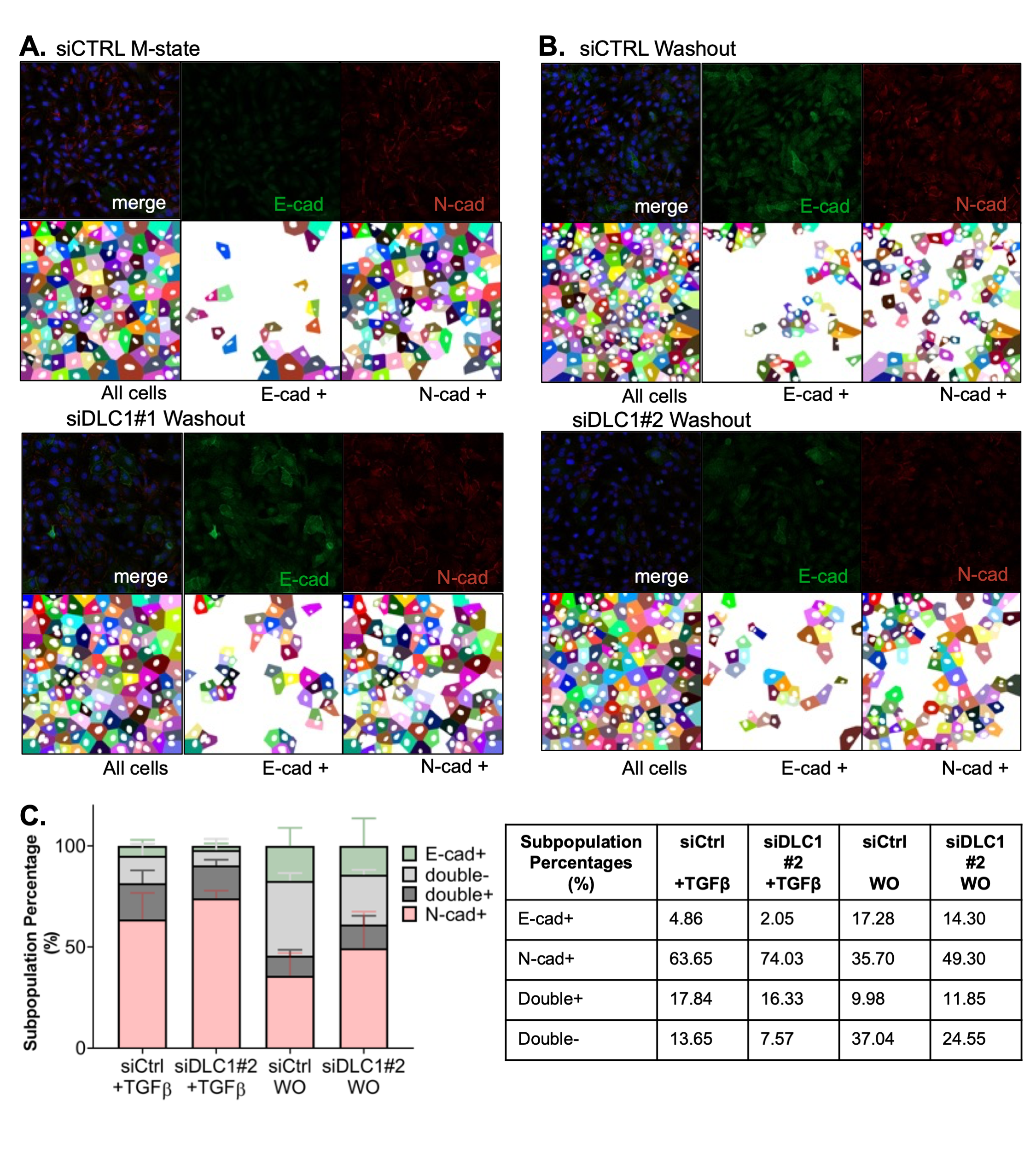

Supplement: S12 Fig — A. IF images using plasma membrane staining of E-cad (green) and N-cad (red) markers for the M-state siCtrl (top) and its representative classification images (bottom). B. IF images using plasma membrane staining of E-cad (green) and N-cad (red) markers for the washout siCtrl, siDLC1#1 and siDLC1#2 (top) and their representative classification images (bottom) in late-EMT using a FIJI script (N-cad + ; E-cad + ; double+ or double-). C. Stacked bar graphs for the average subpopulation percentages of three biological replicates for siDLC1#2 (left). Subpopulation percentages were calculated by the total number of cells per condition (500–1000 cells/condition, right). Two-way ANOVA (Tukey’s multiple comparisons test for siCtrl vs. siDLC1#2 in each subpopulation) was calculated for M-state (+TGFβ) condition: E-cad+ (0.8714), N-cad+ (0.4337), double+ (0.9102) and double- (0.4130); and for washout (WO) condition: E-cad+ (0.8566), N-cad+ (0.2476), double+ (0.8653) and double- (0.0353). (TIF) [file pcbi.1013076.s014.tif]
